# The Role of Cytochrome P450 Holoenzyme Metabolism in the Origin of Neuropathologies

**DOI:** 10.3390/ijms27156971

**Published:** 2026-08-03

**Authors:** Snježana Štambuk

**Affiliations:** Faculty of Forensic Sciences, University of Split, Ruđera Boškovića 33, 21000 Split, Croatia; sstambuk@unist.hr

**Keywords:** cytochrome P450, CPR, adrenodoxin reductase, ALAS1, iron, neuropathology

## Abstract

The increased activity of δ-aminolevulinic acid synthase-1 (ALAS1) leads to the accumulation of δ-aminolevulinic acid (ALA), which may reduce the iron(III) retention ability of ferritin, resulting in iron(III) overload. The accumulation of ALA occurs in the condition of attenuation of the heme negative feedback loop over ALAS1 activity in concert with the induction of *ALAS1* expression. Attenuation of the heme negative feedback loop is maintained by elevated quantities of the heme catabolizing enzyme, heme oxygenase-1 (HO-1), which, in turn, may be induced by highly increased heme concentration. The upregulation of brain HO-1 occurs in patients with Alzheimer’s and Parkinson’s diseases. A mouse with overexpressed human *HMOX1* is a model of schizophrenia with concurrent iron overload. HO-1 inhibitors reduce oxidative damage to whole cells and mitochondrial compartments of rat astrocytes transfected with the *HMOX1* gene. Iron reduction within the heme prosthetic moiety of P450 cytochromes in microsomes is facilitated by NADPH-cytochrome P450 oxidoreductase (CPR), while adrenodoxin reductase performs this function in the mitochondria. In partial CPR-deficient conditions, the half-life of apocytochromes is prolonged while HO-1 is induced due to the elevated heme release from unreduced cytochromes. This study posits that, before being degraded by HO-1, the released hemin triggers the oligomerization of cytochromes, as well as other oxidative damages, by producing hydroperoxyl radicals from hydrogen peroxide produced in uncoupling reaction. After reaching a specific level, iron(III) overload may trigger the saturation of CPR, resulting in the development of neuropathologies.

## 1. Introduction

Heme consists of a porphyrin molecule made of four pyrrolic groups and ferrous ion bound to the center of the porphyrin ring by four nitrogen atoms. A total of 10 to 20% of heme is synthesized in the mitochondria of all the cells, serving mainly as a prosthetic group of cytochrome P450 enzymes (P450s). The remaining 80 to 90% of heme in mammals is synthesized in the mitochondria of the developing erythrocytes with the purpose of being incorporated into hemoglobin. The main heme synthetic enzymes are identical in two pathways of heme synthesis, except for the initial enzyme δ-aminolevulinic acid synthase (ALAS). The first enzyme in heme synthesis required for P450 assembly is a δ-aminolevulinic acid synthase-1 (ALAS1) that produces ALA in the mitochondria. P450s are involved in the oxidative metabolism and biotransformation of a variety of endogenous and exogenous compounds, thus contributing to drug excretion or drug activation, the metabolism of vitamin D3, omega hydroxylation of fatty acids, and the metabolism of cholesterol that results in the production of bile acids, steroids such as glucocorticosteroids, and hormones [1]. It was determined that more than 90% of enzymatic reactions involved in the metabolism of all chemicals (general chemicals, natural and physiological compounds, and drugs) are catalyzed by P450 enzymes [2]. Three-fourths of human P450 reactions involving drug metabolism can be accounted for by a set of five P450 enzymes: 1A2, 2C9, 2C19, 2D6, and 3A4. In P450 enzymes, the heme prosthetic group is bound to the protein active site through a coordination bond with a conserved cysteine residue. Only in its iron-reduced form does P450 exert its characteristic absorption maximum at 450 nm when saturated with carbon monoxide (CO).

Iron reduction in the heme prosthetic moiety of P450 cytochromes in endoplasmatic reticulm (ER) is facilitated by cytochrome P450 reductase (CPR), while in the mitochondria it is led by adrenodoxin reductase. During iron reduction by CPR in P450 cytochromes, some H_2_O_2_ is produced in the uncoupling reaction [3]. Moreover, it has been determined that the production of H_2_O_2_ is increased in the condition of partial inhibition of CPR activity [4].

Heme is degraded by heme oxygenase-1 (HO-1) activity, which is known to be increased in Alzheimer’s disease (AD) and schizophrenia [5,6,7,8]. HO-1 requires CPR for its activity. ALAS1, the rate-limiting enzyme in heme synthesis, is repressed by heme through several different mechanisms. This study suggests that HO-1 may keep the heme negative feedback loop attenuated upon ALAS1 activation through heme degradation, resulting in ferric iron overload due to ALA accumulation that is known to induce the release of iron(III) from ferritin [9,10,11,12,13].

This study argues that in partial CPR deficiency and partial adrenodoxin reductase conditions, where the heme moiety of some P450 species is unreduced while HO-1 activity and the production of H_2_O_2_ by the uncoupling reaction of some P450 species is induced, the P450 species are oxidized by HOO^•^ produced by oxidation of H_2_O_2_ by hemin. In such conditions, cytochrome P420 (P420) species are produced by oligomerization through the oxidation of sulfhydryl groups to disulfide bonds in P450 species, hence releasing the heme moiety more easily. In the same conditions, HO-1 is induced due to increased heme release, allowing the cell to produce an increased quantity of ALAS1. Increased quantities of iron(III) in cytoplasm further prevents frataxin from donating iron(II) to ferrochelatase (FECH). In such conditions, it is possible that mitoferrin provides FECH with iron(III) to facilitate the binding of iron(III) to protoporphyrin in the mitochondria, thus producing hemin instead of heme. The increased quantity of hemin further saturates CPR activity and induces oligomerization of P420 species due to the production of HOO^•^ species that are the source of damaging particles in humans. Therefore, this study argues that the specific regulation of P450 protein metabolism is an underlying cause of some neurodevelopmental, neuropsychiatric, and neurodegenerative diseases, i.e., neuropathologies.

## 2. Acute Porphyrias and Induction of ALAS1 Expression

**Figure 1:**
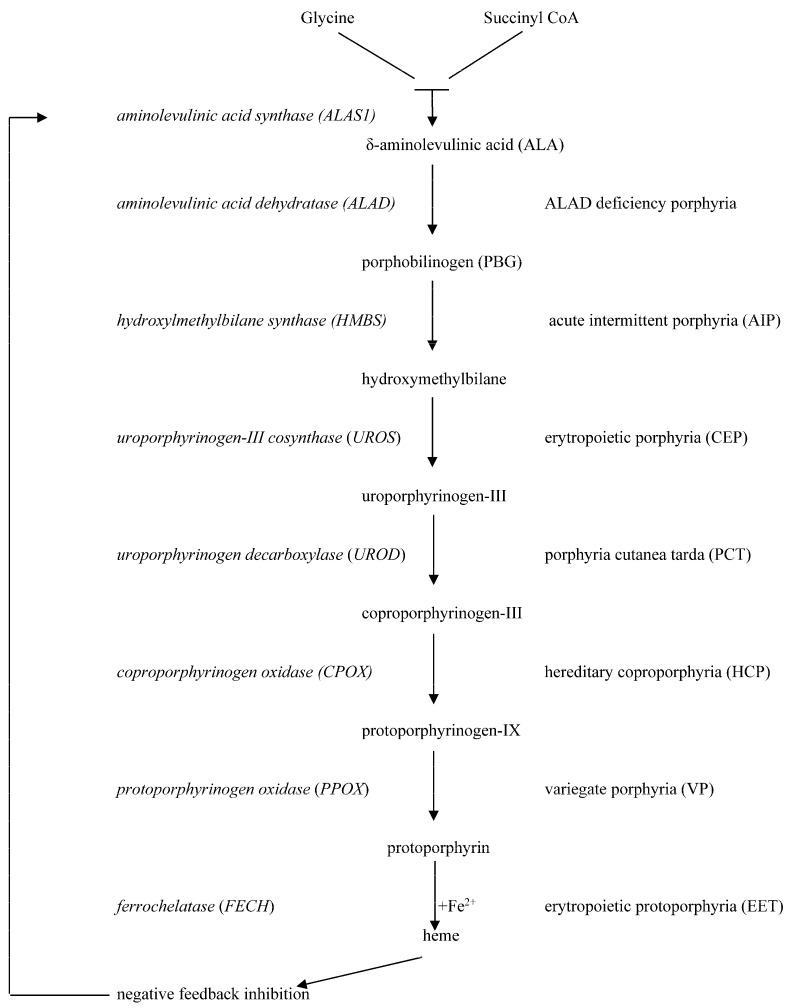
**The pathway of heme synthesis in nucleated cells.** The enzymes that are involved in heme biosynthesis are listed on the left side of the diagram while the types of porphyria that are caused by the respective enzyme deficiency are presented on the right side. ALAS1, PPOX, and FECH are situated in the mitochondria while all the other enzymes are situated in the cytoplasm.

Acute porphyrias are a group of disorders caused by partial dysfunctions in one or more of the enzymes that are members of the heme synthesis pathway in liver (Figure 1) [14].

There are four acute porphyrias: acute intermittent porphyria (AIP), variegate porphyria (VP), hereditary coproporphyria (HCP), and ALAD-deficiency porphyria (ADP). Acute porphyrias may be induced by drugs that cause mechanism-based inactivation of P450. Acute porphyrias are characterized by acute attacks implying autonomic dysfunction, resulting in acute abdominal pain, tachycardia, hypertension, intestinal dysmotility, neuropsychiatric disturbances, and motor neuropathy characterized by quadriparesis. An acute porphyria may fatally end up in death due to respiratory failure caused by the absence of mechanical ventilation.

The known common prerequisite to all types of acute porphyria is the induction of ALAS1 enzyme activity in hepatic mitochondrial compartments and subsequent accumulation of ALA in the liver and plasma [15]. Depletion of the heme pool, which is characteristic of acute porphyrias, permits induction of *ALAS1* expression and activity, since heme is a negative regulator of ALAS1 [16]. The plasma concentration of ALA reaches 10 µM in acute AIP patients [17] and 5 µM in lead-poisoned individuals, compared with 0.1 µM in healthy individuals [18]. Lead inhibits δ-aminolevulinic acid dehydratase (ALAD) (Figure 1). When ALAS1 is highly induced, excretion of ALA is readily detected, indicating that the ability of ALAD to metabolize excess ALA is limited. Liver transplantation in AIP patients with recurrent and incapacitating attacks results in rapid normalization of ALA and abrupt remission of attacks [19]. Through silencing of hepatic ALAS1 mRNA expression in mice using siRNA, the plasma ALA content was significantly decreased biochemically, thus preventing induced acute attacks caused by AIP [20]. Importantly, in the same study, silencing of hepatic ALAS1 protected mice from neuromotor decline provoked by the induction of acute attacks.

The rationale for this study originates in some symptoms in common to acute porphyrias and some neuropathologies. Porphyrias with *ALAD*, *HMBS*, *CPOX*, or *PPOX* deficiencies (Figure 1) are very rare autosomal recessive porphyrias, since only a few cases, proved via mutation analysis, have been described in the literature [21,22,23,24,25,26,27,28,29]. They may include severe developmental retardation, neurological defects, cataract, psychomotor retardation, ataxia, and convulsions, as well as skeletal malformations, most frequently of hands [30]. Recessive acute hepatic porphyrias manifest in infants or in early childhood.

Significantly elevated urinary concentrations of different types of porphyrins have been commonly observed among some children with autistic spectrum disorder [31,32,33,34,35]. At least 53% and possibly more children with autistic disorder excreted excess porphyrin in their urines [32]. The administration of dimercaptosuccinic acid, an agent that removes heavy metals, resulted in a significant drop in urinary porphyrin excretion in patients with autistic disorder. Autism disorder and pervasive developmental disorder might be predicted using porphyrin measures [36].

## 3. ALA-Induced Iron Release from Ferritin, Lipid Peroxidation, and Mitochondrial Damage

Bechara’s lab provided evidence that after prolonged administration of ALA to rats there was an increase in total iron (68%) and ferritin (71%) in the cortex, ferritin in the striatum (44%), protein carbonyls in homogenate of the cerebral cortex (threefold), and ^45^Ca^2+^ uptake by cortical synaptosomes (45%) [37]. Under normal conditions, iron is absorbed from dietary resources through the intestines. An excess amount of cellular iron is stored in ferritin. Cytosolic ferritin stores up to 4500 iron atoms [38]. Iron is transferred into the mitochondria where it serves as a substrate for the synthesis of heme [39] and Fe-S clusters [40]. ALA triggers the release of iron from ferritin in the plasma of uremic patients and in vitro in a dose- and time-of-exposure-dependent manner [9,10]. Moreover, in an in vitro study, an increase in the amount of iron released in an ALA-dependent fashion was observed with an increase in human ferritin level [9]. The total oxygen consumption in vitro was increased about eight-fold in the presence of horse spleen ferritin and 5 mM ALA in comparison with the treatment with 5 mM ALA alone, and it was dramatically inhibited by desferal, an iron-chelating agent [11]. Treatment with ALA was found to alter the primary, secondary, and tertiary structures of the ferritin molecule in a concentration-dependent manner, thus preventing iron sequestration by ferritin [12]. The detrimental activity of ALA decreased tryptophan fluorescence and partially depleted thiols in apo-ferritin. Importantly, iron is kept in ferric Fe(III) form in ferritin [13]. In vitro, ALA had a subtle effect on lipid peroxidation [41], while after the addition of ferritin there was a significant increase in lipid peroxidation in liposomes [10]. Plasma ALA and lipid peroxidation positively correlated, appearing in significantly higher quantity in hemodialyzed uremic patients than in healthy subjects [9].

ALA-inflicted damages to the inner membrane of isolated rat liver mitochondria, such as disrupted mitochondrial membrane potential and induced permeabilization of the inner mitochondrial membrane, were reversed with the addition of iron chelator *o*-Phenanthroline, catalase or superoxide dismutase (SOD), or dithiothreitol (DTT), a disulfide reductant, at the beginning of the collapse of the mitochondrial electrical potential [42,43], and completely reversed with the addition of ethylene glycol tretraacetic acid (EGTA), a metal chelator, even after the collapse [44].

ALA exerted stimulatory effects on Ca^2+^ uptake by cortical synaptosomes, resulting in the inhibition of mitochondrial transmembrane potential and of oxygen consumption [45]. Intrasynaptosomal mitochondrial transmembrane potential diminished in the presence of extrasynaptosomal Ca^2+^ without the additional ALA or in the presence of only the resting Ca^2+^, together with the addition of extrasynaptosomal ALA, and was restored in the presence of ruthenium red, a mitochondrial Ca^2+^ uniporter (MCU) inhibitor. It has been shown that blockage of MCU could alleviate iron accumulation and the associated injury following subarachnoid hemorrhage [46], pointing to MCU as a portal for brain mitochondrial iron uptake. Interestingly, the probability of the opening of MCU is activated by increased intracellular Ca^2+^ concentration [47,48]. Ruthenium red restored the mitochondrial transmembrane potential, even with 2 mM ALA and 1 mM Ca^2+^ [45].

In the presence of Ca^2+^ and ALA, protein aggregates were induced in the membrane of isolated mitochondria, which led to the destruction of mitochondrial transmembrane electrical potential and mitochondrial swelling [49]. Aggregates were also induced in the presence of Ca^2+^ and hydroperoxide or in the presence of Ca^2+^ and diamid, an oxidant of vicinal membrane sulfhydryl groups [50]. The addition of EGTA (which prevents the formation of protein aggregates if combined with ALA treatment) 3 min after collapse did not have a significant effect on the amount of protein aggregates. DTT completely prevented the elimination of transmembrane electrical potential in isolated mitochondria when added in concert with Ca^2+^ and ALA, but was ineffective in prevention when added during the onset of a decrease in transmembrane electrical potential [49]. Apparently, the production of protein aggregates due to thiol cross-linking is sufficient to permeabilize the membrane in concert with the presence of Ca^2+^ [50].

The dysfunction of mitochondrial energetic metabolism has been shown in a mouse model of AIP [51]. The administration of ALA to the human HepG2 cell line induced mitochondrial membrane hyperpolarization and the alteration of the mitochondrial structure and respiration with an increase in reactive oxygen species (ROS) production [52]. Upon ALA exposure of cultures of Schwann cells and peripheral neurons, there was an increase in protein carbonylation, lipid peroxidation, and mitochondrial membrane depolarization accompanied by a reduction in myelin formation levels [53].

The long-term high consumption of iron by rats caused iron overload by increasing plasma non-transferrin bound iron and brain iron levels in a time-dependent manner [54]. Brain lipid peroxidation and brain mitochondrial dysfunction due to increased brain mitochondrial ROS production, as well as brain mitochondrial depolarization and brain mitochondrial swelling, were detected in rats in a time-dependent manner after consuming high levels of iron. Chronic brain iron overload led to impaired brain synaptic plasticity as well as to learning and memory deficits of rats.

Apparently, iron overload, lipid peroxidation, and mitochondrial damage represent the interplay among cellular ferritin, iron, and ALA concentrations. This study argues that the resulting changes in iron homeostasis may trigger damage characteristic of acute porphyrias but also of other neuropathologies.

## 4. Mechanisms of Acute Porphyria Induction: Heme Depletion

**Figure 2:**
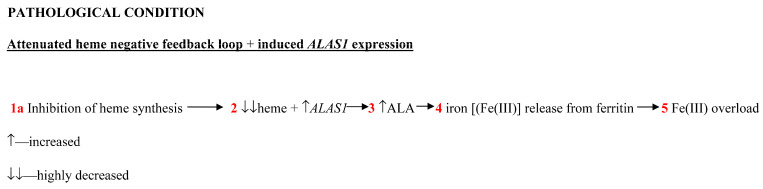
**Pathological condition of inhibition of heme synthesis.** Pathological condition induced via the attenuation of the heme negative feedback loop plus induction of *ALAS1* expression; 1a—inhibition of heme synthesis; 2—heme is low; 3—ALA synthesis is induced; 4—iron is released from ferritin; and 5—iron(III) is overloaded.

Acute porphyrias may be triggered by the partial inhibition of enzymes involved in heme synthesis in liver, except by the inhibition of ALAS1. It is important to emphasize that free heme content is tightly regulated by the rate of its synthesis, insertion into apo-P450 in order to be shielded, and by the rate of its degradation. In turn, hepatic ALAS1, the rate-limiting enzyme in heme synthesis, is repressed by heme through several different mechanisms [16]. Hepatic *ALAS1* is regulated via negative transcriptional feedback by heme in rat liver [55]. Moreover, the inhibition of the transport of the ALAS1 precursor protein into the mitochondria could be triggered by heme binding to the nascent precursor protein in the cytosol of rat liver and mouse liver cells [56,57]. In addition, heme has been shown to decrease ALAS1 mRNA stability in the primary cultured hepatocytes of chick embryo and a chicken hepatoma cell line [58,59], as well as in rat hepatocyte cell lines [60]. In addition, hemin produces conformational change in the mature ALAS1 protein in the mitochondria, thus inducing the degradation of ALAS1 by the action of mitochondrial LONP1 and ClpXP proteases in human liver cells [61,62]. Hence, in order for ALAS1 to be expressed, stabilized, and transported into the mitochondria where the ALA product is synthesized, the heme pool should be depleted. The enhancement of *ALAS1* gene expression in concert with the depletion of the regular heme pool amplifies ALAS1 activity in rats, chick embryo hepatocytes, and mice, all in in vivo conditions [63,64,65]. Hence, the partial inhibition of heme synthesis leads to induction of ALA production in the presence of *ALAS1* expression induction. In these mechanisms of acute porphyrias, the occurrence of the heme negative feedback loop is attenuated, which may result in ALA accumulation under the condition of the induction of *ALAS1* expression [66] and, consequently, as this study suggests, it may also result in ferric iron overload due to iron release from ferritin (Figure 2; 1–5).

In addition, when the heme prosthetic group is alkylated at one of its pyrrole nitrogens, it leaves the P450 active site and is replaced by fresh heme that undergoes repeated N-alkylations, as in the case of allylisopropylacetamide (AIA) treatment [67], representing the mechanism-based inactivation of P450, which also results in acute porphyria.

## 5. Nuclear Receptors, Induction of Heme, and P450 Synthesis

Many apo-P450s are simultaneously synthesized on membrane-bound polysomes and inserted into the membranes of the endoplasmic reticulum (ER) [68,69]. Lipophilic chemicals such as phenobarbital (PB), a potent porphyrogenic drug, upregulate the synthesis of apo-P450 [70] and activate *ALAS1* transcription [64,71]. PB mediates the activation of cytosolic constitutively active receptor (CAR), an orphan nuclear receptor, resulting in its translocation into the nucleus, whereupon it heterodimerizes with another nuclear receptor, retinoid X receptor (RXR). The PB-activated CAR-RXR heterodimeric complex interacts with phenobarbital-responsive enhancer module (PBREM) in the 5′-promoter region of *CYP2* genes, thereby inducing their expression through enhanced transcriptional–translational activation [72,73]. Nuclear receptors, the DNA-binding proteins involved in the activation of *P450* genes, include the pregnane xenobiotic receptor (PXR), CAR, RXR, the peroxisome proliferator-activated receptors (PPARs), the aromatic hydrocarbon receptor (AhR), and the farnesoid X receptor (FXR). They are activated upon binding to their specific ligands that either belong to xenobiotics or endobiotic steroids. The ligand-binding domain of orphan nuclear receptors allows ligand molecules of different sizes to bind with different affinities and transactivating potencies. In order to produce a significant increase in *P450* transcription, a drug has to reach the hepatocyte nucleus in concentrations high enough to stimulate nuclear receptors. Levels of CYP3A4 activity and expression vary up to 60-fold [74]. In vitro PXR/CAR-activation capacity is shown by several porphyrogenic mechanism-based P450-inhibitors, such as tamoxiphene, ritonavir, gestodene, etinylestradiol, mifepristone, and bergamottin.

The induction of the transcription of *CYP3A4* and *CYP2C9* results in a higher *ALAS1* response than the induction of other P450 enzymes. The *ALAS1* gene contains a transcriptional enhancer sequence, the *ALAS1* drug-responsive enhancer sequence (ADRES), containing PXR and CAR recognition sites, which are located 16 and 20 kb upstream of its transcription start site in humans [75]. Hence, lipophilic drugs may stimulate *ALAS1* transcription through xenobiotic-sensing nuclear receptors [76,77]. Other nuclear receptors, steroidogenic factor-1 (SF-1) and liver receptor homolog-1 (LRH-1), which are transcription factors that in concert (SF-1/LRH-1) bind to steroidogenic promotors, also upregulate *ALAS1* gene transcription in steroidogenic cells via binding to a 3.5 kb upstream region of *ALAS1* [78]. Moreover, human hepatic *ALAS1* is a direct target of the bile acid-activated nuclear receptor FXR [79]. Experiments in primary human hepatocytes and in human liver slices showed that ALAS1 messenger RNA (mRNA) and its activity were increased upon exposure to chenodeoxycholic acid (bile acid), the most potent natural FXR ligand, or the synthetic FXR-specific agonist GW4064. Moreover, the overexpression of a constitutively active form of FXR further increased ALAS1 mRNA expression. Binding of FXR/RXR heterodimers to the *ALAS1* 5′ flanking region was demonstrated. Elevated endogenous bile acids may provoke neuropsychiatric attacks in patients with acute hepatic porphyrias [79]. Moreover, some elements in the 5′ region of *ALAS1* mediate the control of the *ALAS1* transcriptional response in the presence of estrogen and ERalpha [80]. An increase in ALA by up to five-fold was achieved by the addition of various C-19 and C-21 steroids to liver cell homogenates, an effect that is dependent on de novo synthesis of the new protein [81]. Pregnane and its derivatives were particularly efficient in inducing ALAS1 activity. However, in some cases, despite the lack of lipophilic chemicals, ALAS1 activity was increased, as shown in chick hepatocyte cultures and rat brain after treatment with succinylacetone, and less so with N-methyl protoporphyrin, ALAD, and FECH inhibitors, respectively [82]. Therefore, this study suspects that ALAD deficiency may have a strong inducing effect on ALA accumulation.

## 6. HO-1, Iron Overload, and Mitochondrial Dysfunction in Neuropathologies

**Figure 3:**
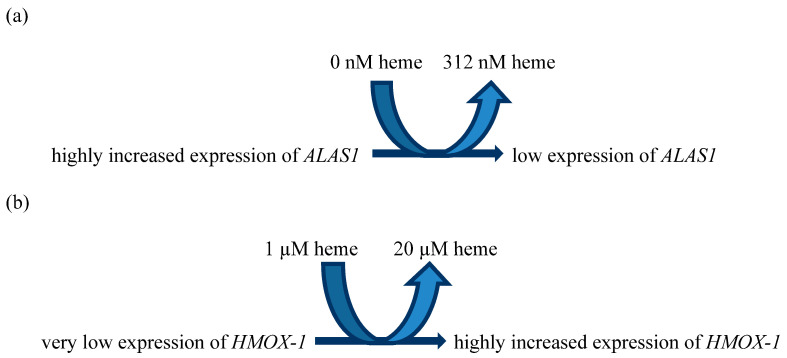
**Expression of *ALAS1* and *HMOX-1* as a function of added heme.** Heme was added to the cell culture of immortalized rat hepatocyte cell lines 3 h before harvesting in gradually increasing concentrations, with starting and finishing concentrations as indicated [60]. The influence of added heme on (**a**) *ALAS1* and (**b**) *HMXO-1* expression.

Heme may be destroyed under the action of HO-1 that produces biliverdin IXa, CO, and free ferrous iron. Biliverdin IXa is reduced to bilirubin using a soluble enzyme, biliverdin reductase. Bilirubin is glucuronidated and then excreted in the bile, CO is released, and iron undergoes reutilization. Heme acts on HO-1 gene (*HMOX-1*) transcription by increasing its rate [83], only slightly, by stabilizing its mRNA turnover. The heme added to the cell culture of the immortalized rat hepatocyte cell lines in concentrations above 78 nM progressively decreased the ALAS1 concentration by acting through a negative feedback loop, thus minimizing ALAS1 synthesis due to the heme concentration approaching 1 μM [60] (Figure 3).

If the cellular heme concentration is increased above 5–10 µM, the concentration of *HMOX-1* starts reaching its maximum, induced by highly increased heme content (Figure 3) [60]. When heme synthesis is abolished in cultured chick embryo hepatocytes, the administration of heme triggers the induction of HO-1 protein [84].

**Figure 4:**
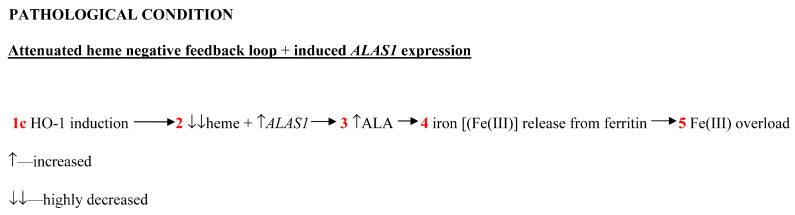
**Pathological condition of induction of HO-1 activity.** Pathological condition induced via the attenuation of the heme negative feedback loop plus the induction of *ALAS1* expression; 1c—HO-1 is induced; 2—heme is low; 3—ALA synthesis is induced; 4—iron is released from ferritin; and 5—iron(III) is overloaded.

Patients with acute porphyria attacks may be treated with heme infusions to prevent attacks [85,86]. In *Hmbs*^−/−^ mice treated with PB and repeatedly with heme arginate, heme catabolism and ALAS1 protein expression were induced while the ratio of reduced vs. oxidized glutathione (GSH) levels was decreased compared with *Hmbs*^−/−^ mice treated with PB but without heme arginate treatment and this was observed coincidentally with HO-1 induction, i.e., the induction of the *HMOX-1* gene and HO-1 protein expression [85]. In the same study, iron deposition was observed as a result of heme arginate treatment. In addition, the correlation between serum ferritin concentration and the number of doses of heme arginate was observed. The same increases in HO-1 protein level, ALAS1 protein level, and in the level of iron deposits were observed in the liver of recurrent AIP patients who received frequent hemin administration compared with the controls [85]. Apparently, an increase in the heme level prevents acute attacks, but if administered repeatedly, it results in the induction of the expression of *ALAS1*, which is probably due to HO-1 induction. This study suggests that HO-1 may maintain attenuation of the heme negative feedback loop upon ALAS1 activation through heme degradation, resulting in ferric iron overload due to ALA accumulation (Figure 4; 1c–5).

Indeed, the treatment of acute porphyria patients with a combined therapy of heme arginate and tin protoporphyrin, a competitive inhibitor of HO-1, resulted in lower excretion of ALA than during the treatment of patients with heme arginate alone [87].

Increased hepatic heme content in mice, due to liver-specific knockout of the gene responsible for cytoplasmic heme export (which regulates intracellular heme content in hematopoietic lineages), induced HO-1 activity and triggered iron accumulation associated with induced lipid peroxidation, while keeping the total cellular heme content as low as it was in the control mice, as was revealed via liver analysis [88]. In the same study, the addition of hemin to the same mice produced increased quantities of the H and L chains of ferritin compared with the control mice. Sustained HO-1 activity in the cell culture increased the expression of *ALAS1*, the level of mitochondrial heme, the concentration of cellular O_2_^• −^, and redistribution of iron from the cytoplasm to the mitochondria [89].

The inhibition of HO-1 completely prevented infection-associated iron increase [90]. In the same study, ferritin was upregulated. In heme-deficient human astrocytoma cells, iron was shown to accumulate due to the corruption of heme synthesis [91] and possible induction of ALA synthesis due to induced ALAS1 activity in heme-depleted conditions. In cultured rat astroglia, *HMOX1* upregulation induced mitochondrial iron deposition, promoted the opening of mitochondrial permeability transition pore, and induced oxidative mitochondrial damage, while HO-1 inhibitors abrogated mitochondrial iron trapping [5,92,93,94,95]. An upregulation of HO-1 mRNA and HO-1 protein has been reported in the cerebral cortex, hippocampus, and microvasculature of the cerebral cortex of patients with AD, but not in the cerebellum, which is free from pathological alterations [6,95], and it co-localized to senile plaques, neurofibrillary tangles, and corporea amylacea [96,97,98]. The overexpression of *HMOX1* in the brain of transgenic mice triggered iron loading-induced tau phosphorylation, resulting in tau aggregation and cognitive decline [99,100]. Astroglial HO-1 promoted mitochondrial damage and biogenesis of corpora amylacea in the intact brain of persons with a mild cognitive impairment but also in the case of *HMOX-1* transfected in astrocyte cultures [101]. An inhibitor of HO-1 administered to rat astrocytes transfected with the *HMOX1* gene reduced oxidative damage to whole cells and mitochondrial compartments [102]. In the same study, the HO-1 inhibitor also attenuated behavioral deficits and neuropathological alterations in transgenic mice, a model of AD. An increase in mitochondrial size and a decrease in mitochondrial number in AD neurons were reported [103]. High levels of somatic mtDNA mutations and mitochondrial abnormalities and damage are prominent features of AD, Parkinson’s disease (PD), and schizophrenia [104,105,106]. Mitochondrial dysfunction occurred early in the development of AD [107]. In PD, *HMOX1* was markedly overexpressed in astrocytes of the substantia nigra and it was also found in Lewy bodies in affected dopaminergic neurons [5,108,109]. The accumulation of iron, besides being a contributing factor in the development of disorders such as AD and PD [110,111,112,113], has also been observed in Huntington’s disease [114,115], multiple sclerosis [116], and amyotrophic lateral sclerosis (ALS) [117]. The selective upregulation of *HMOX1* in astrocytes in conditional transgenic *HMOX1* mice resulted in symptoms similar to those found in schizophrenia and other neurodevelopmental conditions, such as oxidative mitochondrial damage, macroautophagy, and corporea amylacea formation [8]. Importantly, the upregulated *HMOX1* transgenic mice exhibited enhanced astroglial iron deposition in the hippocampus and other subcortical regions, as well as oxidative mitochondrial damage and macroautophagy [7]. The mean content of iron in the caudate nucleus was increased in schizophrenia patients, as revealed in postmortem studies [118]. In addition, in a normal senescent human brain, robust HO-1 painting has been noted in the cytoplasm of ependymocytes of choroid plexus epithelial cells, in cerebrovascular endothelial cells, and within many glial and extracellular corporea amylacea [5]. Moreover, *HMOX1* and ferritin gene expression were significantly increased in the cerebral cortex of aged individuals, as evidenced by postmortem measurements [119].

Interestingly, HO-1 induced by 5 μM hemin exerts increased cell survival rate and anti-apoptotic protein expression, as well as decreased apoptotic cell ratio and apoptotic protein expression, while HO-1 induced by 30 μM hemin triggered decreased cell survival and increased apoptosis [120].

## 7. Free Hemin Exerting Oxidative Action as a Primary Trigger of Neuropathologies

It is unknown how iron triggers neuropathologies. It has been shown that free iron applied to cultured mouse astrocytes was not toxic, while hemin was highly toxic to astrocytes [121]. Furthermore, it has been shown that heme bound to amyloid β, which is a critical pathological feature of AD, exerts the peroxidase activity that may lead to neurotransmitter oxidation [122,123] and/or to amyloid β fibrillation [124]. This study suggests that hemin in interaction with H_2_O_2_ is a primary trigger of neurological damage in all neuropathologies, which is explained further below. Repeated intravenous administration of heme hydroxide (hematin), in doses as low as 4 mg/kg, to monkeys, triggered decreased concentration and activity of P450 [125]. The same effect of lowering P450 activity was achieved by a large single dose of heme arginate (20 mg/kg) [126,127]. Multiple large doses of heme arginate treatment caused the induction of HO-1 with consequential release of iron in rats and dogs [128]. This study posits that HO-1 induction keeps heme low and causes the induction of ALAS1 and consequential accumulation of ALA that acts on iron release from ferritin. The difference in critical doses between hematin and heme arginate may be due to the poor stability of hematin [129,130,131] and relatively high stability of heme arginate. This study suggests that hematin causes a decrease in the concentration and activity of P450 due to the release of unreduced heme (hemin) from P450, and further, free hemin-triggered oxidation of cysteine ligands in P450s, which are then prone to shielding and fast releasing of hemin, as documented below. Indeed, hemin triggers an inverse relationship between the induction of HO-1 and the P450 content in a dose-dependent manner [132].

FECH, which catalyzes the transfer of ferrous iron to the heme moiety, is the last enzyme in the heme synthesis pathway [133]. Although ferrous iron is a substrate for human FECH, ferric iron may also be inserted in protoporphyrin using FECH. Frataxin catalyzes ferrous iron delivery to FECH in the mitochondria [134,135]. The reduction in frataxin due to genetic mutation causes Friedreich’s ataxia, which is a disease affecting the central and peripheral nervous systems. Symptoms of Friedreich’s ataxia consist of gait and limb ataxia, sensory loss, dysarthria, dysphagia, pyramidal signs, cardiomyopathy, skeletal abnormalities, and diabetes mellitus [136]. Histological studies revealed the occurrence of mitochondrial iron accumulation in the cells of Friedreich’s ataxia models [137]. The increased expression of *HMOX-1* has been shown in conditional frataxin knockout mice [138]. In cardiac tissues in a frataxin deficiency mouse model, an increase in the expression of mitoferrin-2 was shown [138]. In a Drosophila model of Fridreich’s ataxia, the downregulation of mitoferrin-2 improved many of the characteristics of the frataxin-deficient condition, including nervous system degeneration, whereas its overexpression exacerbated most of them [139]. This study argues that mitoferrin-2 may donate ferric iron to FECH.

As suggested, either heme synthesis inhibition or HO-1 induction in concert with induced *ALAS1* expression permit the accumulation of ALA, which may act on ferritin stability, causing the release of iron(III). This finding indicates that the increased concentration of iron(III) may be the substrate for FECH, thus resulting in the elevated production of hemin, which is then incorporated into P450 protein. This study posits that iron in hemin is reduced with hydrogen peroxide, producing ferrous iron and hydroperoxyl radicals:

hemin(Fe^3+^) + H_2_O_2_ → heme(Fe^2+^) + HOO^•^ + H^+^

It is reasonable to suggest that hydroperoxyl radicals oxidize P450 axial cysteines, which results in P450 oligomers bound with disulfide bonds. P450 oligomers are prone to shielding and release of hemin due to weaker ligands bound to hemin. It is possible that the oxidation of the thiol group of axial cysteines in P450 is induced by exceeding the level of surplus free hemin in relation to the free protein domain of P450 species, which results in the formation of P420 species with weaker axial ligands in P420 protein; these species are prone to faster release of hemin than unoxidized P450 proteins. Hydrogen peroxide is a by-product of P450 catalytic activity due to uncoupling reactions [3]. Indeed, it has been shown that P450 undergoes inactivation in liver microsomes of untreated PB and 3-methylcholantrene-treated rats and rabbits; moreover, it was found that this inactivation was dependent on H_2_O_2_ produced during the uncoupling reaction triggered by P450 CPR reduction [140,141]. It is important to emphasize that CPR-dependent P450 reduction even occurs in conditions without a substrate present [142]. The process of P450 self-inactivation, which is dependent on H_2_O_2_ produced in the P450 system, is accompanied by heme loss, P450 protein aggregation, and loss of SH-groups in P450 [141]. Apparently, covalent aggregation of P450 molecules is a later process than the process of heme release.

In conclusion, released hemin interacts with hydrogen peroxide and produces hydroperoxyl radicals that subsequently oxidize cellular components, triggering different pathologies as a result of cellular ferric iron overload.

## 8. Partial CPR Deficiency: Genetic Studies

All microsomal P450, HO-1, and cytochrome b_5_ reductions depend on CPR for the supply of electrons required for their activities. CPR is a diflavin reductase that uses NADPH as an electron donor [143]. NADPH must first reduce CPR before CPR delivers electron(s) to P450. The first electron from CPR reduces the ferric heme to its ferrous state in the heme prosthetic moiety of P450. When CO was added to microsomes that had been treated with a reductant, i.e., dithionite [144], it produced a ferrous–CO complex with a Soret absorption band at 446 nm [145]. P450 cytochromes bind CO only when they are in reduced ferrous form.

In a mouse model of liver-specific deletion of the *CPR* gene, hepatic microsomal CPR expression was significantly reduced at three weeks of age as well as two months of age in heterozygous (for approximately 50%) and homozygous *CPR* mutants (approximately 82% at three weeks and up to an undetectable level at two months) [146]. While P450 activities were significantly decreased in all mice, HO-1 activity was unchanged in both heterozygous mice compared with wild-type mice, while it was below 5% in both homozygous mice, the feature ascribed to CPR deficiency, i.e., to deficient HO-1 reduction. It is possible that HO-1 binds to CPR with greater affinity than P450, which explains the unchanged HO-1 activity in heterozygous mice, while decreased activities of P450 were found in the same mice. The total P450 protein content and P450 mRNA expressions were increased by several fold in both homozygous mice. Importantly, HO-1 protein level was increased nine-fold in 2-month-old homozygous mice. Homozygous liver-specific *CPR* mutants were fertile and normal in gross appearance and growth rate.

*CPR* knockout mice (*CPR-null* mice) are embryonically lethal and characterized by neural tube, cardiac, eye and limb abnormalities or by generalized retardation of development several days after gestation [147]. Importantly, in the given study, *CPR* knockout mice were positive for CPR protein, as was revealed via immunoblot analysis. CPR-truncated protein lacking the membrane-binding domain was detected only in cytoplasmic cellular fraction but not in microsomal fraction. Embryonic lethality was observed in heterozygous germline *CPR-null* mice. Moreover, there was no greater lethality for the *CPR*^low/low^ fetuses than for the *CPR*^low/+^ fetuses, with both amounting to 40% fetus lethality [148]. CPR-low mice had 70–90% suppression in the levels of *CPR* expression in all organs examined, including the brain, which had a 90% decrease. Adult CPR-low mouse expressed astrocytosis in the subventricular zone [149]. Astrocytosis is a characteristic of AD [150] and it was shown to occur in schizophrenia [151]. This study argues that partial CPR deficiency might be involved in the development of different neuropathologies, depending on the extent of CPR deficiency.

Antley–Bixler syndrome, which may be influenced by *CPR* mutation, presents itself at birth or prenatally. Features of the disorder include serious skeletal deformities [152] but also renal, cardiac, and visceral malformations. The skull and long bone deformities of Antley–Bixler syndrome were recapitulated via conditional *CPR* deletion in mice [153]. In a family with genetic *CPR* deficiency, subnormal activities of some P450 species were observed despite genotyping data predicting their normal or high enzymatic activities [154]. Six out of seven patients with CPR deficiency, whose baseline serum ACTH and cortisol were mainly in the normal range, failed to mount appropriate cortisol response to ACTH stimulation [155], as is the case with schizophrenia [156,157] and in patients with the first episode of psychosis [158]. Patients with schizophrenia have lower cortisol levels than controls, both in anticipation and after exposure to social stress [159].

## 9. Heme Release and HO-1 Induction in Partial CPR-Deficient Conditions

HO-1 is induced in CPR-deficient conditions in mice [146]. In selenium-deficient microsomes, CPR activity has been reported to be about half of the activity found in selenium-proficient microsomes [160]. In selenium-deficient rats under PB treatment, the activity of HO-1 in liver is rapidly and markedly stimulated (eight-fold) [161,162], pointing to accelerated heme degradation. Indeed, the excretion of ^14^CO in the breath after administration of ALA[5-^14^C] was greater by PB-treated selenium-deficient rats than by similarly PB-treated controls [162]. In PB-treated selenium-deficient rats, accelerated turnover of heme was detected in cytochromes, which was measured by the drastic temporal disappearance of ^14^C-heme in rat liver cytochromes 8 h after treatment with a pulse label of δ-[5-^14^C]ALA [161]. Pretreatment with selenium an hour prior to PB treatment lowered the PB-mediated stimulation of HO-1 activity by as much as 36%, while pretreatment for 4 or 6 h essentially abolished the PB-mediated stimulation of HO-1 activity.

Depletion of circulating thyroid hormone in rats via hypophysectomy or treatment with the anti-thyroid drug methimazole led to a 75–85% depletion of hepatic microsomal CPR activity and protein level [163]. Hypophysectomy in rats caused the concentration of holo-P450 in microsomes to decrease to an almost spectrally undetectable level as measured at 450 nm, although the protein moiety of cytochromes did not decrease; on the contrary, it slightly increased, as detected in acrylamide gel containing SDS [164]. The decrease in P450 content was due to the absence of the heme moiety in P450. In hypophysectomized rats, HO-1 activity was induced. Importantly, a three–four-fold greater heme transfer to human serum albumin from microsomal preparations was obtained from the hypophysectomized rats in comparison with the control rats, pointing to possible facilitated heme release from P450 from microsomes in hypophysectomized rats. In the same study, it was shown that the depressed synthesis of heme, enhanced degradation of heme, or decreased concentration of protein moiety in microsomal membranes were not responsible for the enhanced loss of holocytochrome P450.

It has been shown that CPR decreased by 50 to 75% in liver microsomes isolated form hypophysectomized rats and that restoration of P450 reductase levels could be achieved via treatment with T4 [165]. Triiodothyronine (T3) increased CPR mRNA in human adrenal and liver cells [166,167]. Interestingly, P450 content was decreased via T3 administration, which was determined by measuring the protein moiety content of P450; HO-1 activity remained uninduced, and the incorporation of ^3^H-ALAS1 into the liver microsomal heme was markedly reduced compared with thyroidectomized rats [168]. In addition, the affinity of the P450 protein moiety with heme was not decreased in the presence of T3. Hence, heme release is more pronounced in CPR-deficient conditions than in the presence of CPR.

Therefore, in CPR-deficient conditions, HO-1 was induced and P450 decreased when measured at 450 nm, which is due to the absence of the heme moiety while the protein moiety was stable. It is very important to pinpoint that this only partial deficiency in CPR is required for HO-1 activity and the production of H_2_O_2_ (during uncoupling) involved in iron-driven P450 oxidation and consequent shielding and release of heme. Hemin is toxic for cellular constituents in the presence of hydrogen peroxide due to the production of hydroperoxyl radicals.

## 10. Influence of CPR Activity on P450 Half-Life

A hepatoma cell culture study demonstrated that diphenylene iodonium (DPI), a partial inhibitor of CPR, induced an almost seven-fold increase in CYP2E1 half-life, amounting to 26 h, compared with CYP2E1 half-life without the addition of partial CPR inhibitor, while the total cellular protein content remained unaffected in the presence of the partial CPR inhibitor [4]. In that study, CYP2E1 content was measured with antibody against CYP2E1 in an inhibition of cellular protein synthesis condition. In the same study, it was shown that partial inhibition of CPR activity induced the production of H_2_O_2_. In hypophysectomized rats, the levels of cytochromes designated as P450b and P450e were 10–50 fold elevated compared with intact adult male rats, while T3 administration completely reverted this effect [169].

On the other hand, a study demonstrated that upon the expression of human *CPR* in Chinese hamster ovary cells transfected with human *CYP2D6*, the level of CYP2D6 was reduced to 30% of the level found in cells without recombinant *CPR*, which was determined by measuring the peak at 450 nm, while CYP2D6 activity was several-fold greater [170]. In addition, the suppression of human CYP2E1 protein moiety content in concert with an increase in human CPR content occurred in insect cells transfected with *CYP2E1* and *CPR*, which was determined via Western blot analysis, i.e., by measuring the content of CYP2E1 protein moiety [171]. On the contrary, CYP2E1 activity increased upon co-expression with *CPR* in the same experimental condition. Upon co-expression of *CPR* in Chinese hamster ovary cells transfected with *CYP3A4*, the enzyme activity of CYP3A4 was increased 15-fold, despite a 40% decrease in spectrally active CYP3A4 at 450 nm, which did not occur as a consequence of decrease in CYP3A4 mRNA [172].

In hypophysectomized rats, the content of different P450 species increased several fold, while treatment with T3 reduced P450 species to less than 10%, which was revealed by using antibodies against them [165,173]. At the same time, the same study showed that the relative activity of those P450 species decreased in hypophysectomized rats, which was reversed up to the control level with CPR or thyroxine (T4) administration to liver microsomes. T4 administration to hypophysectomized rats augments CPR activity in liver microsomes. Hence, in the presence of proficient CPR, the P450 half-life is shorter and P450 activity is greater compared with the condition of deficient CPR, where the half-life of at least the protein cytochrome moiety of unreduced iron containing cytochrome species is longer, while the P450 activity is weaker. Moreover, those results point to the reduced state of P450 in the absence of a substrate, which is contrary to the common belief that their reduction occurs only after binding of the substrate. Apparently, the P450 protein moiety of unreduced iron containing cytochrome species is more viable than that of reduced iron containing P450 cytochromes, but they release the heme moiety more readily than reduced iron containing cytochromes, thus inducing HO-1 activity.

The above-described results point to the detrimental effect of unreduced hemin on P450 activity due to the partial lack of CPR, as well as the enforced release of heme, which in turn results in HO-1 induction.

## 11. CPR Saturation: Biochemical Studies

The pretreatment of rats with drugs such as PB altered the profile of P450 enzymes and their susceptibility to inhibition by parathion [174]. Parathion inhibited P450 enzymes by reversible and irreversible mechanisms. Potent reversible inhibition of CYP2B1 by parathion occurred in PB-induced fractions, probably as a consequence of saturation of CPR since a P450:CPR ratio of about 30:1 has been reported after PB treatment. This result points to the possibility of mutual competition among P450 enzymes during binding to the limited amount of CPR. Treatment of human hepatocytes isolated from different liver samples, which were placed into primary culture with 1 mM PB, resulted in an 18-fold increase in CYP2C8 mRNA, a six-fold enhancement of CYP2C9 mRNA, a 23-fold increase in CYP3A4 mRNA, and a 10-fold increase in CYP2C19 mRNA content [175]. Moreover, treatment of chick hepatocytes with PB and PB-like drugs was found to increase HO-1 activity [84], which probably points to enhanced heme release as a consequence of CPR saturation. In microsomal membranes of rodents, CPR is expressed at a much lower quantity than P450 content, amounting to 1/10th to 1/20th of the P450 concentration [176], while in human liver samples, this ratio is in the range of 1:2–1:27 with an average level of 1:7.1 [177]. In untreated rats, CPR is present in excess of HO-1, with a HO-1:CPR ratio of about 0.2:1. Treatment of rat liver microsomes with cadmium, which led to the induction of *HMOX1*, increased the HO-1:CPR ratio to 3:1 [178], which is close to 2:1, the point of maximal activity of HO-1 [179].

In a CPR saturation condition, one should assume the existence of P450 species that are lacking heme, which occurs due to heme release, which oxidizes cysteine ligands in P450 and causes their oligomerization. Indeed, with the administration of greater quantities of PB or 3-methylcolanthrene (MC) alone to rats, the amounts of P450(PB-1) and P450(MC-1) cytochromes without a bound heme were calculated to be 20–30% of the total P450(PB-1) and P450(MC-1) content, which was measured by determining the level of radioactivity of the heme moiety with [^14^C-ALA] after its dissociation from the P450(PB-1) and P450(MC-1) species previously immunoprecipitated from rat liver microsomes [180]. PB and MC treatments may trigger CPR saturation due to highly increased P450 synthesis. CPR saturation should result in HO-1 induction. Indeed, PB and MC treatments induce HO-1 activity [84,181].

## 12. P450 Oligomers Produced via Thiol Oxidation

The tendency of P450 to form oligomers in solution and in membranes is well known [182,183,184,185,186,187,188,189,190]. It has been shown that Cys^239^ residues of two subunits of CYP3A4 oligomers were brought in close proximity to each other after the treatment of CYP3A4 with thiol-reactive bifunctional reagents [191]. In the same study, the interaction of P450 in the second type of the inter-subunit interface was also likely to bring some cysteine residues other than the Cys^239^ of two CYP3A4 subunits into close proximity to each other. Higher order P450 aggregation of CYP1A2 was associated with the state of inhibition of CPR reduction in P450, while monomers are prone to reduction in correlation with CPR concentration [192]. Indeed, oligomerization of microsomal P450 in the membrane has been shown to decrease upon their interaction with CPR [193,194] and upon reduction in P450 [184]. In heteromeric cases as for CYP2B4 and CYP1A2, the metabolism of CYP1A2 was stimulated, while that of CYP2B4 was inhibited by 30–50% when the CPR concentration was subsaturating [195,196]. Once CPR concentration exceeded the total P450 concentration, the rates of metabolism of substrates by the mixed P450 aggregates equaled the sum of the rates by the individual P450 species [195]. The condition of P450 aggregation apparently favors metabolism by one species in aggregates while other species are unfavorable to CPR reduction at a subsaturating concentration of CPR. This model fits the observation that highly favored metabolization by one member of P450 aggregate produces H_2_O_2_ via the process of uncoupling. However, all other members of aggregate that are unreduced by CPR shield and release hemin, which subsequently triggers the oxidation of cellular components after being reduced by H_2_O_2_, thus producing hydroperoxyl radicals that previously triggered P450 oligomerization.

## 13. Appearance of a Peak at 420 nm Provoked by Hemin Administration

Reconstitution of functional holocytochrome P450 with added heme to liver homogenate from rats treated with PB and AIA produced a highly increased absorbance peak at 420 nm, which is characteristic of P420 species [197]. Those P420 species are reduced by DTT to P450 species [197]. Once P450s are detached from microsomes, they form oligomers and are characterized by a peak at 420 nm, which represents the solubilized form of P450 species [198,199]. Those enzymatically inactive forms are also isolated as ferric species of P450, but with a characteristic absorption band at 420 nm when saturated with CO [199,200]. The conversion of P420 to P450 may proceed using sulfhydryl compounds, such as cysteine, homocysteine, GSH, and thioglycolic acid, while the conversion of P450 to P420 may occur using urea, acetone, deoxycholate, and *p*-chloromercuribenzoate (*p*-MB) [199]. It is assumed that P420 species are forms of P450 that have lost the characteristic thiolate ligand in cysteine, which is coordinated with heme by replacing it with a thiol group [201]. This study posits that P420 are formed by the oxidation of the thiolate group of cysteine that is coordinated to heme in P450 species, which results in the creation of a disulfide bond and the transfer of the coordination to another amino acid in apocytochrome. Indeed, the replacement of the axial cysteine with histidine in P450cam showed its Soret maximum at 420 nm [202]. In the same study, it was shown that histidine’s electron donation to the heme iron is weaker than that of cysteine. In addition, it has been shown that when CO binds to the ferrous P420, a histidine ligand is recruited as the heme ligand [203].

In experiments using P450_cam_ from *Pseudomonas putida*, titration of the freshly acid–acetone-prepared apoprotein, which, once bound with heme gives rise to P420 forms, yielded 4.5 sulfhydryl residues compared with six sulfhydryl residues obtained after the action of dithioerythritol over the same apoprotein [204]. Holo-P450_cam_ contains 6 moles of p-MB-titrable sulfhydryl groups. Interestingly, in in vitro reconstitution of apoprotein to holo-P420 with heme to protein ratios less than 1, the formation of P450_cam_ was proportional to the heme concentration, while ratios above 1 of heme–apoprotein favored P420 over P450_cam_. Moreover, the addition of heme to native P450_cam_ yielded additional cytochromes with P420 spectral properties without affecting the total amount of P450_cam_ present.

The addition of acrolein to rat lung or liver microsomal suspensions resulted in partial conversion of P450 to P420, which was reversed with the addition of GSH or DTT [205]. Moreover, added acrolein triggered the inactivation of CPR. Acrolein treatment caused approximately 40% and 28% decreases in protein sulfhydryl content in lung and liver preparation, respectively. This study posits that under acrolein treatment, P420 species appeared due to oxidation of the axial cysteine of the thiol group into the disulfide bond in cytochrome as a consequence of CPR inactivation. Indeed, it has been shown that GSH may act upon the conversion of P420 to P450, which is required within the cell to prevent the formation of aberrant disulfides [206]. In addition, deoxycholate converted 80% of P450 to P420 during 8 h of incubation at room temperature [199]. It has been suggested that cholate seems to act by breaking P450-CPR complexes and solubilizing the phosphatidylcholine membrane [207].

## 14. Enhanced Release of Heme from P420 Forms in Comparison with P450 Forms

While the transfer of heme from the membrane-bound or solubilized preparations of P450 to human albumin and rabbit apohemopexin was found to be nonexistent, the transfer of heme from P420 to both species readily occurred [208]. Hence, heme is readily released from P420 species but not from P450 species. Indeed, the inactivation (bleaching) of P420, which is probably due to heme release, occurred in both P420 oligomers (about two-thirds are involved in bleaching) and P420 monomers (intense bleaching) [209]. In the same study, it was concluded that oligomerization resulted in a considerable stabilization of the cytochrome. Hence, the protein moiety of those P420 forms is more stable and their half-life is possibly longer than that of forms with a thiol group, but they release the heme moiety more promptly than P450 species. Indeed, after prolonged treatment of rats with microsomal preparations of PB and 3-amino-1,2,4-triazol (a partial inhibitor of δ-aminolevulinate dehydratase), a decreased binding of heme with P450 was observed [210]. Moreover, it has been shown that heme exchanges reversibly between different molecular species of P450 in livers of PB- or MC-treated rats [211], and it turns over faster than its protein moiety [212].

## 15. The Molecular Effect of Partial CPR Deficiency: A Hypothesis

**Figure 5:**
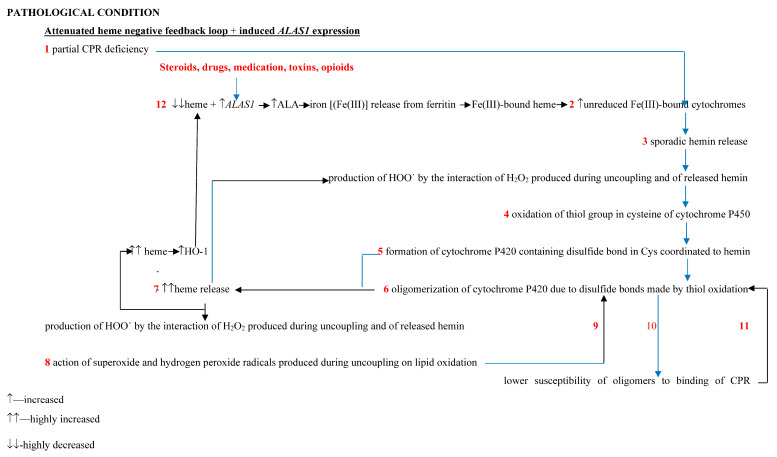
**Pathological condition of partial CPR deficiency or CPR saturation.** Pathological condition induced via the attenuation of the heme negative feedback loop plus induction of *ALAS1* expression; 1—partial CPR deficiency or saturation; 2—unreduced ferric cytochromes due to partial CPR deficiency; 3—heme is released sporadically; 4—oxidation of the thiol group in cysteine; 5—formation of P450 containing disulfide bonds; 6—oligomerization of P450 by disulfide bonds produced by thiol oxidation; 7—hemin release is enhanced; production of HOO˙ by the interaction of H_2_O_2_ produced during uncoupling and of hemin; 8—lipid oxidation is induced via an uncoupling reaction due to the production of superoxide radicals and less so by the production of hydrogen peroxide; 9—oligomerization of cytochromes enhanced by lipid peroxidation; 10—oligomerization-induced lower susceptibility of cytochrome oligomers to binding of CPR; 11—further oligomerization due to inadequate binding of CPR; and 12—HO-1 depletion of heme and consequent iron(III) overload.

**Figure 6:**
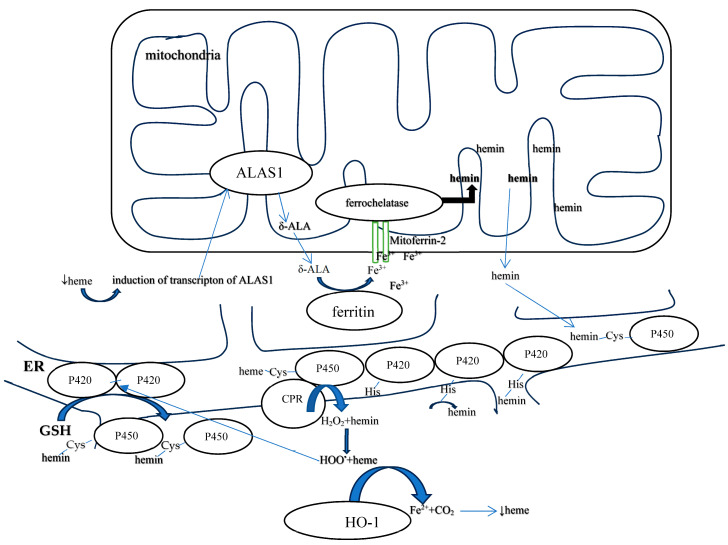
**CPR protein is saturated**. The picture shows what is happening in the cell when the ratio of CPR proteins vs. P450s proteins is low. In the beginning of CPR saturation hemin oxidizes cysteine coordinated to hemin in the P450 species producing P420 oligomers with hemin bound to histidine. Such species are prone to release of hemin that further oxidizes peroxide produced in the uncoupling reaction between reduced P450 and CPR, in that way producing hydroxyl radicals. Hydroxyl radicals further oxidize P450 species resulting in the oligomerization of P420 proteins with prolonged half-life but with the characteristics of lousy binding of hemin. Protein HO-1 keeps low quantity of heme in the cell. Low concentration of heme in the cell induces transcription of ALAS1 protein that produces δ-ALA that in its turn causes release of oxidize iron (Fe^3+^) in cytoplasm. Fe^3+^ enters the mitochondria through mitoferrin-2 that transfers Fe^3+^ to FECH that synthetizes hemin. Increased quantities of Fe^3+^ further saturate CPR protein in vicious circle resulting in the P420 oligomer synthesis. GSH reduces disulfide bonds in P420 producing P450 monomers.

This study posits that partial CPR deficiency, saturation of CPR activity, or partial inhibition of CPR activity or mutation in *P450* in the CPR-binding site may be the triggering cause of oxidation of thiol groups in cytochrome’s cysteines via interactions of sporadically released hemin and hydrogen peroxide, which results in the oligomerization of cytochromes (Figure 5, 1–6; Figure 6).

**Figure 7:**
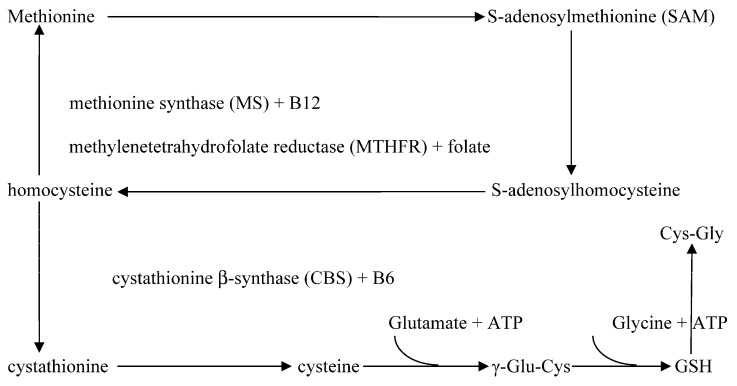
**Methionine pathway and GSH synthesis**. MS is involved in the synthesis of methionine from homocysteine, which requires vitamin B12 for this action. In addition, MTHFR is involved in methionine synthesis, which requires folate for its action. CBS in concert with B6 catalyzes the conversion of homocysteine in cystathionine. GSH is synthesized from cysteine.

**Figure 8:**
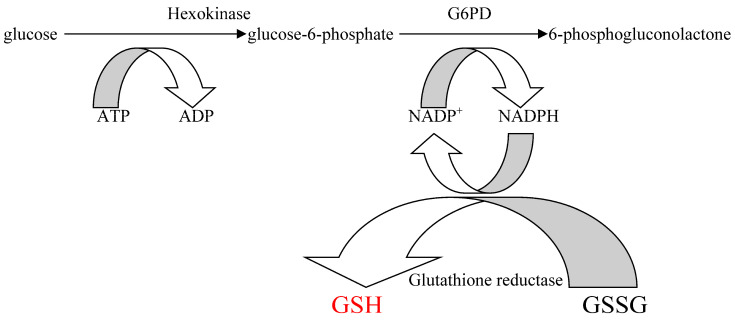
**The pathway of GSH synthesis via glutathione reductase**. The cellular synthesis of GSH using NADPH produced in pentose phosphate pathway as a cofactor in glutathione reductase reduction in glutathione disulfide (GSSG), the oxidized form of GSH.

Indeed, the partial loss of heme leads to the formation of oligomers [182]. If microsomes are incubated aerobically for 20 h, both protoheme and P420 disappear, while P450 species fall to 73% of their initial content, as measured via spectroscopy [198]. In anaerobic conditions, all three species remain at the same level as before incubation. This study suggests that heme is probably sporadically released from P450 species in an aerobic condition due to ferric heme. Hydrogen peroxide released in an uncoupled reaction of P450 reduction by CPR produces hydroperoxyl radicals in the interaction with free hemin and further oxidizes –SH groups in P450, resulting in the production of P450 oligomers (Figure 5 and Figure 6). This study posits that P450 oligomerization may result in the formation of P420 forms via the oxidation of thiol group(s) into disulfide bonds in cysteine to which heme is coordinated, which results in the creation of cytochrome oligomers that are less prone to CPR action (see below and Figure 5). Cytochrome oligomers containing disulfide bonds in axial cytosine are susceptible to hemin release upon binding it, resulting in HO-1 induction (Figure 5, 7; Figure 6). This study further hypothesizes that the released hydroperoxyl radical exerts oxidative action, primarily over lipids but also over other cellular species (Figure 5; 8), which produces damage that may result in neuropathologies, depending on the proficiency of the repair mechanisms of the oxidative damage. Released heme is subject to heme degradation by HO-1 in cytoplasm and the mitochondria, thus keeping the heme negative feedback loop attenuated and producing ALA accumulation in the case of concomitant induction of *ALAS1* expression (Figure 5 and Figure 6). Accumulated ALA produces iron(III) overload through the action on ferritin. This study suggests that iron(III) overload saturates CPR activity and triggers hemin release that, in concert with hydrogen peroxide, produces hydroperoxyl radicals that oxidize axial cysteines producing P420 species. Indeed, ALA treatment, both of a mouse with mutation in a liver-specific gene responsible for cytoplasmic heme export and of primary hepatocytes isolated from that mouse, resulted in an increase in heme content, lipid peroxidation, and HO-1 and ROS production [88]. In addition, iron increase has been associated with the induction of *HMOX1* in the neurons, microglia, and capillary endothelial cells of rats [90].

## 16. Appearance of the Peak at 420 nm Provoked via ALA Administration

Added ALA in concentrations above 8 × 10^−5^ M to rat hepatocyte culture caused an increase in heme content as well as an increase in the 420 nm peak, while the peak at 450 nm remained at the same height up to the concentration of 10^−4^ M ALA, as measured using the dithionite-reduced CO difference spectrum [213]. At concentrations of 10^−4^ M ALA, there was twice more P420 species than P450 species, as measured with the dithionite-reduced CO spectrum. The rise in ALA concentration up to 8 × 10^−5^ caused a gradual rise in peaks at 450 nm. Hepatocytes in vivo react to the administration of ALA by increasing the synthesis of heme while keeping the concentration of spectrally active P450 at the same level [214]. By measuring the incorporation of [^55^Fe] into heme, [^55^Fe]heme increased four- to seven-fold in the presence of ALA (1 × 10^−4^ M) [213]. This study posits that increased content of ALA induces CPR saturation and released hemin amplifies a vicious circle of HO-1 induction and ferric iron release via ALA action over ferritin (Figure 5, 2-3-4-5-6-7-8-2; Figure 6). Increased heme content [213] and, as this study suggests, induced HO-1 due to CPR saturation, as well as iron overload and consequent iron(III) release caused by ALA accumulation, may all have occurred in ALA-treated cells. Moreover, the oral administration of ALA in concert with ferrous iron (which is prone to oxidation in cellular conditions) induced HO-1 expression in peripheral blood mononuclear cells of healthy human individuals, while ALA or ferrous iron alone could not elicit the same effect [215]. Moreover, an increased concentration of ALA in concert with ferric iron triggers the induction of HO-1 synthesis in rat fibroblasts [216].

## 17. Influence of Iron(III) and Other Metal (III) Treatment on Induction of ALAS1 and HO-1 and on Decrease in Heme and P450 Content

In acutely and chronically intraperitoneally iron(III)-treated rats, hepatic HO-1 and ALAS1 activities were increased [217,218,219]. In acutely iron(III)-overloaded rats, P450 content was significantly decreased, as measured using the difference spectrum of P450 between dithionite–CO-P450 (externally reduced P450) vs. CO-P450 at 450–510 nm [217,218]. This study posits that iron in a ferric-bound form in the heme moiety of holocytochrome may trigger CPR saturation. Oral administration of iron to rats strongly augmented the induction of hepatic ALAS1 activity by certain drugs that induce hepatic porphyria [220,221] or by fasting [222]. Indeed, the induction of ALAS1 in chronically iron(III)-overloaded rats was enhanced by PB and AIA [217]. In addition, in chronically iron(III)-overloaded rats, the P450 content significantly decreased in AIA- and PB-treated animals, which was measured as the peak height at 450 nm, and P450 activity was decreased in AIA-treated animals [217]. Long after the rats were treated with iron(III) (up to 84 days), the liver iron concentration remained constant, while there was a persisting slight increase in the activity of hepatic ALAS1 (110–150%) [218], which was probably due to an increase in unreduced cytochrome forms and the subsequent vicious circle of hemin-induced oxidation of P450 forms, hemin release, and HO-1 induction. Indeed, after providing rats with labeled heme and subsequent addition of iron dextran, there was a significant 30% loss of microsomal heme radioactivity compared with the control animals, while HO-1 was increased by 3.3-fold [219]. Moreover, P450 content was decreased in rats treated with iron [223]. This study suggests that after reaching a specific level, iron(III) overload may be an underlying cause of the development of neuropathologies due to its CPR-saturating role.

Some metal ions, such as manganese, ferric iron, cobalt, nickel, and lead, induce HO-1 and ALAS1 activity while decreasing heme content and P450 activity and content, as measured at 450–490 nm [16,217,224,225,226,227,228]. These metal ions did not have stimulatory effects on HO-1 enzyme in vitro. Some of the metal ions mentioned above are inserted into the porphyrin group by FECH but are nonfunctional in holo-P450 structures [16,197,229]. All of these metals may exist in (III) or (IV) oxidative states that make them active in the reactions of oxidation. This study posits that porphyrins with the above-mentioned metals incorporated are involved in the process of oxidation of P450 at the cystein ligand that forms disulfide bonds between P450 oligomers.

The addition of cobalt induces HO-1 activity. The formation of P450 in cobalt-treated rats was twice as low as in the control animals, which was measured via the peak surface at 450 nm [213]. This process was not dependent on the inhibition of heme synthesis but rather on the enhanced heme breakdown, which was probably due to the induction of HO-1 activity by cobalt. Moreover, in the same study, it was shown that a decrease in the total quantity of heme in rats treated with cobalt was due to a decrease in heme in P450 forms extracted from microsomes. This decrease points to greatly enhanced heme release from cytochromes. Cobalt- and ALA-treated cultured hepatocytes, when acting together, produce a highly increased peak at 420 nm and a decrease in the peak at 450 nm when compared with the condition of ALA treatment (10^−4^ M) only. Again, the peaks were obtained using the dithionite-reduced CO difference spectrum. Hence, in ALA-, hemin-, and cobalt-treated conditions, P450 species disappear, while P420 species increasingly appear. Importantly, in the same conditions, P450 activities decrease.

## 18. Appearance of P450 Forms In Vivo

All measurements of P450 and P420 forms are performed in the presence of dithionite as a reducing agent of ferric iron in the heme moiety, which generally appears in in vitro conditions. In an experiment measuring Fe(II)-CO vs. Fe(II) difference spectra of total P450 in human hepatocyte culture, it was revealed that all of the P450 content detected in vivo was found in a reduced state, producing one peak at 450 nm, as detected by comparing the P450 spectra with the added external reductant to the one without it, which is contrary to the common belief that in the resting state, P450s are found in ferric (oxidized) forms [230]. The spectral characterization of P450s is typically performed on P450 enzymes after their extraction from the normal intracellular environment. All of the expressed human P450s in bacterial cells could be considered as predominantly reduced (ferrous iron) in the presence or the absence of an external reductant [230]. The exceptions were CYP2C9 and CYP3A4, which only in the presence of an external reductant exerted an absorbance peak at 450 nm or at around 420 nm and 450 nm respectively. Otherwise, in bacterial cells, CYP2C9 did not produce a significant peak between 400 and 500 nm, while CYP3A4 produced a small peak at around 420 nm.

Steapsin, which is a crude pancreatic lipase, causes the conversion of CO-binding pigment into P420 forms [198]. Moreover, steapsin digestion appears to remove CPR from microsomes. P420 forms decreased rapidly in isolated CO-binding microsomal particles under aerobic conditions after steapsin action, and this decrease was accompanied by the decomposition of residual protoheme. In these conditions, protoheme and P420 finally amount to less than 30% of their initial content, while P450 amounts to 73% of its initial content [198]. Indeed, under an inert atmosphere, the apoprotein P450 retains the sulfhydryl groups prepared from the native enzyme, while the free sulfhydryl content of the protein decreases slowly upon storage, suggesting that some –SH groups are slowly oxidized to S-S groups [204].

## 19. Lower Susceptibility of P450 Oligomers than That of P450 Monomers to CPR Activity

In the case of heme synthesis being inhibited with the exception of ALAS1 activity, heme being alkylated in P450 as in the case of AIA treatment, HO-1 being induced, or CPR being partially deficient or saturated, this study posits that iron is released from ferritin in ferric form due to ALA accumulation. If the cell lacks the appropriate concentrations of CPR, then iron in the heme prosthetic moiety remains in oxidized form, which may trigger the enhanced oxidation of cytochrome cysteine thiol into a disulfide bond, ending up with the creation of P450 oligomers and further hemin release. It has been shown that electron flow to a fraction of CYP3A4 members of oligomers is hampered and that P450 monomerization of CYP3A4 results in its complete reducibility [231,232]. The reducibility of the high-spin fraction in P450 oligomers has been shown with both the NADPH-dependent reduction and the flavin domain of bacterial P450BM-3, which was used as a soluble substitute for the membrane-bound CPR [233]. On the contrary, the low-spin heme protein that encompasses around 66% of species in oligomers was not reducible in both cases. The same fraction of low-spin oligomeric P450 proteins in solution amounting to 65–70% is susceptible to a pressure-induced P450 transformation to P420 forms and is incapable of binding substrates [209]. Hence, oligomerization may enhance suppression in CPR reduction in cytochromes, creating another vicious circle (Figure 5; 6-10-11).

## 20. Lipid Peroxidation and P450 Oligomerization

Lipid peroxidation decreased P450 activity and rotational mobility, while it increased the formation of protein complexes of high molecular weight in rat liver microsomes, thus pointing to heightened oligomerization of P450 protein [234,235]. Indeed, from human placental mitochondrial and microsomal studies, Klimek reported iron-dependent, reduction-dependent, and uncoupling process-dependent loss of P450 content and activity due to, more prominently, superoxide or, less pronouncedly, hydroperoxide production [236,237,238,239,240,241,242]. This loss of P450 activities negatively correlates with the extent of lipid peroxidation. The loss of P450 activity is a result of the lack of P450 reduction as assigned by observing the loss of the spectroscopic signal at 450 nm [241,242]. In addition, it has been shown that this lipid peroxidation and the loss of P450, as measured at 450 nm, positively correlate with ALAS1 concentration and activity [243]. Again, it is important to emphasize that superoxide and hydroperoxide production during the uncoupling reaction occurs in the absence of a substrate, meaning that the uncoupling reaction happens during CPR-dependent reduction in P450 independently of substrate presence. Hence, by forming another vicious circle, lipid peroxidation provoked by CPR-dependent uncoupling reaction determines the extent of P450 oligomerization (Figure 5; 6-7-8-9-6) and hence, the extent of hemin release.

## 21. Implication of GSH Depletion: Decrease in P450 Content and Activity During Increase in HO-1 Content

Importantly, holo-CYP2B1 reconstitution with the addition of hemin in liver homogenates from AIA-treated PB-pretreated rats was maintained by GSH [244]. Moreover, the catalytic activity of CYP3A4 toward testosterone or nifedipine was strongly stimulated by the addition of GSH in the reconstituted system, which was partially due to the increased affinity between CYP3A4 and CPR and especially between CYP3A4 and cytochrome b5 [245,246]. As suggested, GSH may prevent aggregation by the reduction in cysteine residues in P450 which are involved in disulfide formation [247]. The increase in the transcription of *HMOX-1* was found to be a direct response to GSH depletion [248,249]. The decrease in mitochondrial GSH levels has been associated with mitochondrial swelling and degeneration in rat brain [250]. A deficit of GSH in mice resulted in behavioral, morphological, electrophysiological, and neurochemical alterations analogous to schizophrenia and bipolar disorder [251]. This study posits that the reduction in the disulfide bonds of cysteines in cytochromes by GSH may break disulfide bonds and produce P450 monomers (Figure 6) that are prone to CPR-reducing activity, which is in accordance with GSH-induced enhanced binding between cytochrome and CPR [245] and the GSH role of the heterotropic effector on the decrease in CYP3A4 cooperativity in the microsomal membrane [252]. Indeed, increased bilirubin triggered by cobalt injected into rats was diminished by the addition of GSH or cysteine [224]. Moreover, P450 activity and content and heme content were not decreased in the case of the administration of cobalt, nickel, or platinum with GSH or cysteine, while it was decreased with the addition of cobalt, nickel, or platinum alone or in concert with albumin [224,228]. Hence, the effects of cobalt on bilirubin formation, the decrease in heme content, and the decrease in P450 content were enforced by the depletion of GSH, while they were restored with the addition of GSH [224]. In addition, cadmium-, nickel-, or platinum-provoked induction of HO-1 activity in human cell culture was attenuated by GSH or cysteine [228,253] and weakened by iron chelators [253]. Cadmium increased the level of lipid peroxidation while it decreased the level of GSH [254]. This study posits that the vicious circle of the negative effect of oligomerization on CPR binding to cytochromes is reversed by the action of GSH on the enhancement of P450 monomerization (Figure 6). GSH was markedly decreased in the cells of Friedreich’s ataxia patients as well as in a yeast model of Friedreich’s ataxia [255].

## 22. Decrease in Steroids in AIP

The urinary concentrations of 41 out of 55 steroids analyzed from AIP patients were found to be significantly decreased, and the extent of decrease positively correlated with the degree of hydroxylation [256]. AIP patients with higher concentrations of ALA and porphobilinogen had lower concentrations of steroid and the significance of this negative correlation increased with the number of hydroxylation steps that occurred in biosynthesis of steroid. These results point to decreased enzyme activities triggered by hydroxylation steps during which H_2_O_2_ is produced that may act, as this study suggests, to reduce iron(III) into iron(II). This reaction, this study posits, produces hydroperoxyl radicals that are subsequently involved in the oxidation of P450-forming oligomers as well as of other cellular components.

## 23. Possible Role of Iron(III) in Mitochondrial Damage

It has been shown that iron overload in rats detected as an increase in plasma non-transferrin bound iron and in brain iron caused an increase in brain lipid peroxidation, brain mitochondrial ROS production, brain mitochondrial depolarization, and mitochondrial swelling, all in a time-dependent manner in the condition of high iron consumption [54]. The combination of deferiprone, an iron chelator, and N-acetyl cysteine restored all of the deleterious effects of iron overload as well as learning and memory deficits elicited in rats by iron overload. In addition, the combination of deferipron and N-acetyl cysteine prevented the destruction of the blood–brain barrier and attenuated the expression of apoptosis markers.

It has been shown that mitochondrial cytochrome CYP11A1 probably existed as an oligomer in the mitochondrial liposomal membrane [257], where it induced vesicle aggregation enhanced by cardiolipin and increased protein content over total lipids in the membrane [258]. Vesicle aggregation was abolished by adrenodoxin [258], which otherwise exerts heme iron reduction over mitochondrial P450 by transferring electrons from NADPH through adrenodoxin reductase to the iron of mitochondrial cytochromes of P450 [259]. Adrenodoxin is involved in transferring the electrons to CYP11A1, CYP11B1, CYP11B2, CYP24A1, CYP27A1, and CYP27B1 [260]. Loss of adrenodoxin or adrenodoxin reductase triggered increased mitochondrial iron overload up to 30-fold [261]. Moreover, loss of adrenodoxin triggered an increase in HO-1 protein content. These results possibly point to the same influence of unreduced iron—both in microsomal and mitochondrial cytochromes—on heme release, induction of HO-1 activity, and iron overload. However, in the case of the mitochondrial compartment alone, this influence occurs due to the deficiency of adrenodoxin and/or adrendoxin reductase. It appears that adrenodoxin reductase is limited in placental mitochondria, which determines the rate of progesterone synthesis [262,263]. Apparently, the creation of disulfide bonds causes the formation of oligomers from mitochondrial cytochromes, which prevents the reduction in heme iron via adrenodoxin and adrenodoxin reductase.

Indeed, biallelic mutation in adrenodoxin reductase (ferredoxin reductase) that retains 20% activity of the enzyme causes optic atrophy, ataxia, hypotonia, other neurological anomalies, and respiratory failure [264]. Patient fibroblasts showed mitochondrial dysfunction and increased ROS production. Such defects were rescued by the overexpression of wild-type *FDXR* (ferredoxine reductase gene). In addition, mice carrying the same, most frequent mutation found in patients recapitulated the defects characteristic of patients.

## 24. Detrimental Effects of Hemin

Hemin induces lipid peroxidation, protein thiol modification, and carbonyl formation, causing a progressive decrease in mitochondrial membrane potential, while autophagy is activated upon hemin exposure as a protective mechanism [265]. Hemin in concert with hydrogen peroxide causes extensive oxidative modification of low-density lipoprotein (LDL) in in vitro conditions contrary to free iron added as ferrous or ferric species [266]. Hemin causes the destruction of red blood cell membranes into grain-like structures that depend on hemin concentration and incubation time [267].

## 25. Molecular Mechanisms of Mitochondrial Dysfunction and ER Stress

Mitochondrial injury and depolarization caused the redistribution of cardiolipin from the inner mitochondrial membrane to the outer mitochondrial surface as a signal for autophagy machinery to remove damaged mitochondria [268]. Apparently, faulty autophagy is a contributing factor to AD pathology, since a study of AD brain showed a massive accumulation of autophagic vacuoles within large swelling along dystrophic neurites and defects at different steps of the autophagic–lysosomal pathway [269].

Cadmium led to ER stress in a cell line derived from rat liver and in mice [270,271]. Manganese induced ER stress and mitochondrial dysfunction in neuronal cell culture [272]. ER stress is involved in the pathology of AD, PD, and non-alcoholic fatty liver disease (NAFLD), and in diabetes [273]. Acrolein, which triggers the inactivation of CPR and induces AD-like pathologies in vitro and in vivo in rat brain [274], induces ER stress, mitochondrial dysfunction, GSH depletion, and oxidative stress, which are attenuated by N-acetyl cysteine [275].

Moreover, ER-stress inducers, such as thapsigargin and tunicamycin, have been shown to cause an increase in the expression of *HMOX-1* in fibrosarcoma cells [276]. The addition of either N-acetyl cysteine or GSH to the cell culture before ER stress completely blocked the induction of *HMOX-1* expression and activity. The treatment of vascular smooth muscle cells with brefeldin A or thapsigargin, both of which induce ER stress, as well as with homocysteine that promotes protein misfolding in the ER, caused the enhanced expression of *HMOX-1* [277].

The ratio of reduced GSH to oxidized GSH is greater than >50:1 in the cytoplasm, and 1:1 to 3:1 in the ER lumen [278]. Oxidative protein folding may trigger the accumulation of misfolded proteins or the inability of the protein to attain its proper configuration through the formation of mispaired disulfide bonds [273]. During disulfide bond formation, ROS are generated as a consequence of the oxidation/reduction environment. Importantly, ER stress events amplify mitochondrial ROS production within cells [273]. Prevention of mitochondrial ROS accumulation completely blocks cell death, but not the ER stress response. The condition of the depletion of GSH during ER stress allows ROS to be generated by the mitochondria, while mitochondria-associated ROS may in turn further enhance the ER stress response, thereby amplifying mitochondrial ROS accumulation [273]. Oxidative ER stress causes a Ca^2+^ influx into the cytoplasm from the extracellular environment or from the ER/Sarcoplasmic Reticulum (SR) [273]. An increase in Ca^2+^ concentration in the cytoplasm promotes Ca^2+^ influx into the nuclei and mitochondria. Hence, at the early stage of ER stress, oxidative stress causes the release of Ca^2+^ from ER, and a large portion of the released Ca^2+^ is taken up by the mitochondria, which results in ROS production [273]. Iron can enter hippocampal neurons not only through the voltage-operated calcium channel (L-VOCC) but also through other types of neuronal Ca^2+^ channels. This study posits that calcium channels may also be the entry site for iron in neuropathologies. Indeed, mitochondrial calcium uptake (MCU)-open probability is activated by increased intracellular Ca^2+^ concentration [47,48]. Moreover, high ROS levels in the mitochondria further increase Ca^2+^ release from the ER [273].

In acutely iron dextran-loaded rats, ER stress markers were all increased, while N-acetyl cysteine lowered the levels of these parameters [279]. The presence of the *HFE* H63D mutation (which is associated with cellular iron overload) in the human neuronal cell line, is associated with persistent ER stress [280]. These findings were recapitulated in a transgenic mouse model carrying *Hfe* H63D in the same study.

## 26. Timothy Syndrome: Monogenic Neuropathology

It has been shown that L-VOCC agonists enhance iron influx in neurons [281,282]. Iron can enter hippocampal neurons not only through L-VOCCs but also through other types of neuronal Ca^2+^ channels. Bipolar disorder and schizophrenia patients, carriers of the risk variant SNP rs1006737 in the *CACNA1C* gene, showed increased levels of CACNA1C mRNA in human dorsolateral prefrontal cortex measured from postmortem brain samples [283]. The *CACNA1C* gene, which encodes the alpha-1C subunit of the Cav1.2 voltage-dependent L-type calcium channel, is the only gene known to be associated with Timothy syndrome (TS), which is therefore considered a monogenic neurodevelopmental disorder [284]. TS is a complex disorder that affects multiple organs, characterized by autistic traits, webbed fingers and toes, dismorphic facial features, and immunodeficiency [285]. The mutation that causes this disorder revealed a gain of channel function due to the loss of voltage-dependent inactivation, which resulted in heightened calcium influx [286].

## 27. Type 2 Diabetes Mellitus and Liver Diseases Co-Appearing with Acute Porphyrias and Neuropathologies

In a cohort of psychiatric patients, unexplained sudden deaths were associated with diabetes and dyslipidemia [287]. The prevalence of type 2 diabetes mellitus (T2DM) was significantly increased in schizophrenia compared with the control population [288], and T2DM was the most frequently recorded comorbidity in deceased schizophrenic persons, contributing to 31.4% of hospital deaths [289]. Frequent coexistence of diabetes mellitus and acute porphyria has been reported in humans and experimental animal models [290,291]. T2DM is characterized by persistent high blood glucose associated with insulin resistance and relative insulin deficiency, with the latter being due to the progressive failure of pancreatic β-cells [292]. Pre-diabetes and T2DM have some mechanisms in common with AD, with iron accumulation being one of them [293]. A whole spectra of liver diseases were observed in patients with T2DM, including abnormal liver enzymes, NAFLD, cirrhosis, hepatocellular carcinoma, and acute liver failure [294]. The prevalence of NAFLD in diabetic patients was 58–82% [295]. The most obvious co-appearing features of NAFLD are central obesity, insulin resistance, fasting hyperglycemia, and hypertriglyceridemia [296]. Metabolic syndrome is characterized by features such as hypertension, dyslipidemia, high levels of visceral fat deposition, insulin resistance, higher than normal blood glucose due to impaired glucose tolerance, and T2DM, all of which may be accompanying factors in schizophrenia [297,298,299,300] and bipolar disorder [301]. Insulin resistance and impaired glucose tolerance appeared in antipsychotic-naïve schizophrenia patients [297,298,302,303,304,305], even in the first-degree relatives of patients compared with the healthy unrelated controls [304,306], as well as in drug-free chronic schizophrenia patients [307,308]. A decrease in lactate and an increase in glucose in the cerebrospinal fluid of antipsychotic-naïve schizophrenia patients in comparison with the controls [309], as well as in the urine of antipsychotic-naïve schizophrenia patients (mainly first-onset psychosis), have been reported [310].

## 28. Iron Overload, Mitochondrial Dysfunction, and HO-1 Activity in T2DM and Liver Diseases Co-Appearing with Acute Porphyrias and Neuropathologies

Mitochondria in T2DM β-cells exhibit impaired hyperpolarization in response to glucose as well as morphological and functional abnormalities [311], resulting in mitochondrial dysfunction [312]. Evidence of the link between iron overload and diabetes [313] was found during the observation of individuals with pathologic iron overload, as in hereditary hemochromatosis (HH) [314], transfusional iron overload [315,316], such as bone marrow transplantation [317], and Friedreich ataxia (for a review, see [318]). HH implies the abnormal accumulation of iron in parenchymal organs. The prevalence of diabetes in persons with HH over the age of 45 exceeded 20% [319]. *Hfe*^−/−^ mice, a model of HH, accumulated 72% more iron in the islets of Langerhans and four-fold more iron in the liver, compared with the wild-type control. The mice also exhibited decreased insulin secretory capacity secondary to oxidative stress, decreased glucose-stimulated insulin secretion, and increased β-cell apoptosis [320]. Insulin secretory abnormalities improved when persons with HH underwent phlebotomy [321].

Iron deposition in the liver has been found to induce insulin resistance [322,323,324], resulting in the reduced capacity of the liver to suppress glucose production, leading to hyperglycemia. It has been shown that hepatic iron overload coexists in patients with chronic liver diseases [325,326,327]. Another study demonstrated that patients with liver iron overload almost always displayed insulin resistance irrespective of liver damage [323]. Furthermore, phlebotomy or reduced iron intake normalized insulin resistance and improved liver function in patients with NAFLD [328,329]. Mitochondrial dysfunction has been reported to precede insulin resistance and liver steatosis [330], and it persisted in the pathology of steatosis [331]. Interestingly, a longer duration of hepatic iron exposure in HH increased the risk of developing hepatic fibrosis [332]. Phlebotomy reduced fibrosis in HH-triggered hepatic iron overload [333]. Furthermore, numerous studies have established an association between HH and AD as well as HH and ALS [334].

The induction of *HMOX1* expression by exogenously administrated heme to rats increased oxidative stress and iron accumulation in rat liver, while an inhibitor of HO-1 prevented such increases [335]. An increase in heme concentration level that triggered a high increase in HO-1 mRNA resulted in renal and liver cell necrosis [336]. Increased levels of HO-1 were also found in plasma of the newly diagnosed T2DM [337]. Induced *HMOX1* expression increased malondialdehyde and decreased SOD in rats with hepatic fibrosis [338]. Moreover, lowering of *HMOX1* expression improved rat liver fibrosis in end-stage cirrhosis by reducing iron accumulation [338]. Conditional *HMOX-1* deletion in mice hepatocyte and macrophage reduced steatosis and liver toxicity [339]. HO-1 is one of the strongest positive predictors of metabolic diseases in insulin-resistant obese subjects compared with the healthy population.

Apparently, co-appearance of T2DM and liver diseases with neuropathologies are not random but may be triggered by common mechanisms, i.e., iron overload and consequent mitochondrial dysfunction. Moreover, HO-1 induction is a common event in neuropathologies and co-appearing pathologies, i.e., T2DM and liver diseases. The possible role of some triggering functions of T2DM and liver diseases in the occurrence of neuropathologies is described in the following section.

## 29. Hyperhomocysteinemia and Glucose-6-Phosphate Dehyrogenase Deficiency

Homocysteine is a sulfur-containing amino acid formed via demethylation of methionine (Figure 7) [340].

Methionine is an intermediate in the biosynthesis of cysteine. Homocysteine is remethylated to methionine by the vitamin-B12-dependent enzyme methionine synthase (MS). This reaction also requires the participation of methylenetetrahydrofolate reductase (MTHFR), a folate-dependent enzyme. Through the trans-sulfuration pathway, homocysteine forms cysteine in a reaction catalyzed by a vitamin-B6-dependent enzyme, cystathionine β-synthase (CBS) (Figure 7).

Methionine synthase reductase (MTRR) catalyzes the reductive methylation of MS. Specific polymorphisms in *MTR* (coding for MS) and *MTRR* genes may significantly increase the risk of having hyperhomocysteinemia (HHC) [341]. The reason why the lack of remethylation to methionine might induce HHC lies in the fact that the S-adenosylmethionine (SAM) that is created from methionine, and further recycled back to homocysteine, is the allosteric regulator of CBS [342]. The activity of human CBS increases up to five-fold upon the binding of SAM to the C-terminal region of CBS [343]. Hence, methionine recycling is important for CBS functioning. Under the condition of methionine restriction, the CBS protein level is decreased 10-fold. An increase in hepatic carbon monoxide (CO) content causes a global decrease in trans-sulfuration metabolites such as cystathionine, cysteine, and hypotaurine [344]. HO-1 produces CO that inhibits CBS in a concentration-dependent manner [344,345].

There are two pathways of GSH production: the first pathway is through the cysteine intermediate described above (Figure 7), and the second pathway is dependent on the activity of glutathione reductase, which requires NADPH for its activity (Figure 8).

Importantly, CO also shifted glucose utilization toward the pentose phosphate pathway, thereby providing high NADPH for the sake of the production of GSH [346,347], since the main cellular source of NADPH is the pentose phosphate pathway.

**Figure 9:**
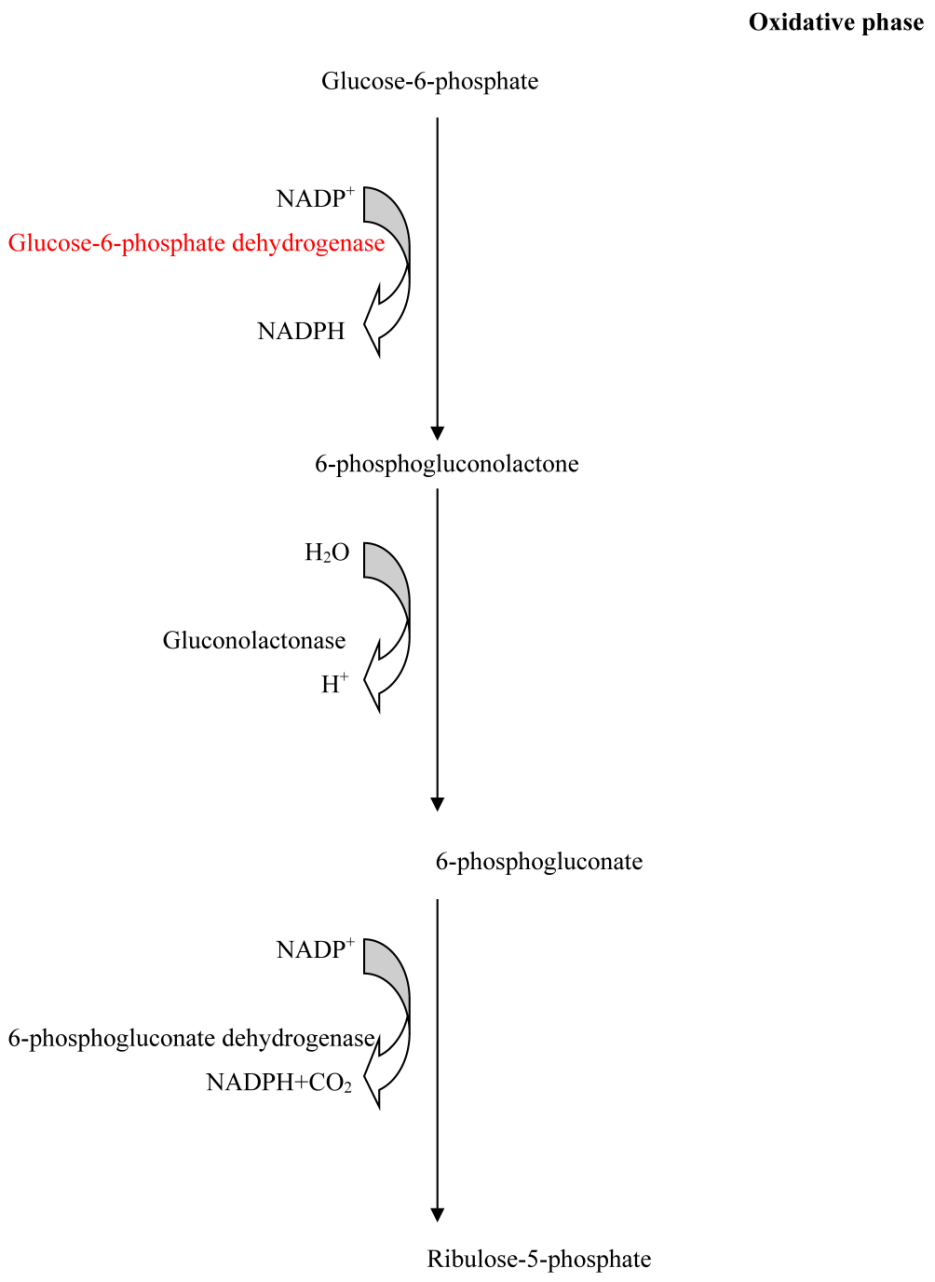
**The pentose phosphate pathway: oxidative phase**. G6PD is the first enzyme in the pentose phosphate pathway that is involved in NADPH production.

The main rate-limiting enzyme in the pentose phosphate pathway is glucose-6-phosphate dehydrogenase (G6PD) (Figure 9).

The diabetogenic effects of streptozotocin, i.e., decreased levels of GSH, may be reversed by the addition of nicotinamide, either in mice or in rats as experimental models [348,349]. G6PD deficiency is associated with hemolysis, which develops after eating certain foods, such as fava beans, or after taking certain medications [350]. Interestingly, while manic schizoaffective disorder is diagnosed in one fourth of the overall population of the studied outpatients, it was excessively represented in the subgroup of G6PD-deficient patients (38%) [351]. In *G6PD*(+/def) mice, enhanced neurodegenerative changes were observed in brain regions exhibiting elevated DNA oxidation, as revealed by histological analysis [352]. Interestingly, *G6PD* knockout clones of embryonic stem cells were extremely sensitive to hydrogen peroxide and the sulfhydryl group oxidizing agent, diamid [353].

**Figure 10:**
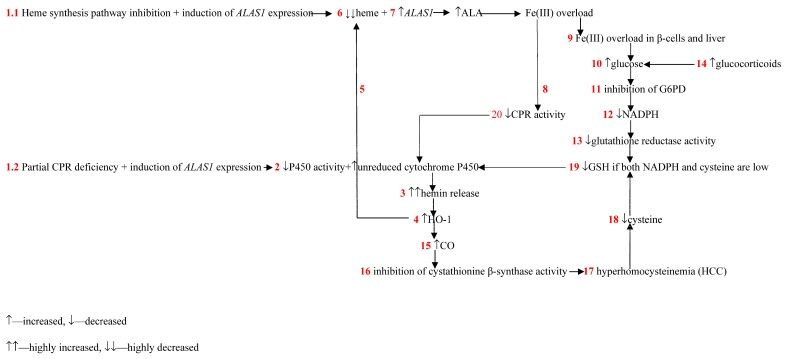
Lowering of CPR activity in case the pathway of heme synthesis is partially inhibited or CPR activity is saturated. Pathological conditions of organisms are characterized by the induction of *ALAS1* expression and by low heme concentrations that are unable to repress *ALAS1* transcription through the heme negative feedback loop due to the following: 1.1—Inhibition of the heme synthesis pathway. 1.2—CPR saturation. 2—Deficient P450 activity due to partial CPR deficiency triggers an increase in hemin release due to an increase in unreduced P420. 3—Increased hemin release. 4—*HMOX-1* expression is induced. 5—Increased HO-1 activity degrades heme, lowering its cellular content. 6—Heme content is decreased. 7—Increased expression of *ALAS1* triggers iron overload. 8—Iron overload increases the oxidation of cysteines in P450 forms due to CPR saturation enabling the formation of cytochrome oligomers that are less prone to CPR activity, resulting in the accumulation of unreduced cytochrome forms. 9—Mitochondrial iron overload in β-cells and hepatic cells may induce hyperglycemia. 10—Hyperglycemia may be induced by mitochondrial iron overload or by an increased concentration of glucocorticoids. 11—G6PD is inhibited by high glucose. 12—G6PD inhibition causes decreased production of NADPH. 13—In the condition of decreased NADPH production, the activity of glutathione reductase is reduced. 14—Increased cellular glucocorticoid content triggers hyperglycemia. 15—HO-1 produces CO. 16—Produced CO inhibits CBS. 17—Attenuation of CBS activity results in HHC. 18—A defect occurs in the production of cysteine required for GSH production due to the attenuation of CBS activity. 19—In the case of the depletion of both the cysteine and NADPH cellular pools, GSH production is decreased. 20—CPR activity is lower. Acting in synergy, both pathological characteristics, hyperglycemia and HCC, may cause an enhanced decrease in cellular GSH. In the condition of decreased GSH, the binding between CPR and P450 forms is lower due to the enhanced formation of cytochrome oligomers. GSH depletion results in lower P450 activities and reduced P450 content due to the lack of proficient reduction in P450 by CPR. The vicious circle of CPR deficiency is triggered by the oxidation of the cysteine(s) of the P450 enzyme with free hemin.

Since CBS inhibition by the CO that is produced by HO-1 may abolish cysteine-dependent GSH production (Figure 10; 4, 15-20), this study proposes that the NADPH cofactor is necessary in the case of the increased demands for GSH reduction activity required for the enhancement of CPR.

The requirement for the NADPH cofactor produced by G6PD activity also occurs in the case of deficiency in any one of the members in cysteine synthesis. Homocystinuria, an inherited disorder of the metabolism of methionine resulting in HHC, is characterized by mental, skeletal, psychiatric, ocular, and arterial disorders [354]. There is a possible link between HHC and schizophrenia [355,356,357,358]. The observed depletion of serum cystine in schizophrenia [310] may be indicative of a compromised GSH system in the brain under increased oxidative stress in neurological diseases. In the same study, it was found that 2-hydroxybutyrate is increased in the serum of individuals with schizophrenia, which is a by-product of the reaction producing cysteine from homocysteine. Importantly, 2-hydroxybutyrate has been found to be an early biomarker for insulin resistance and glucose intolerance in a non-diabetic population [359]. In addition, HHC has been shown to be a co-appearing factor with AD and vascular dementia [360,361,362]. Some AIP patients with peripheral neuropathy have been reported to show elevated homocysteine in plasma [363]. Moreover, patients with NAFLD were found to have increased levels of plasmatic homocysteine compared with the controls [364].

GSH has been found to be less abundant in the liver of *CBS*^−/−^ than in control mice [365]. In addition, the expression of *HMOX-1* is strongly induced in the liver of *CBS*^−/−^ mice at the transcriptional and protein expression levels. It has been shown that the plasma folate concentration was lower in patients with schizophrenia [366], especially those exhibiting HHC [357,367]. Increased plasma homocysteine was found to be negatively associated with plasma folate and vitamin B12 in patients with schizophrenia [367,368]. Moreover, folate deficiency, due to the triggering of HHC, was found to be a risk factor for PD [369]. Folic acid, B12, and B6 reduced homocysteine levels [370]. Vitamin B (folic acid and vitamins B6 and B12) treatment markedly reduced gray matter atrophy in patients with AD by lowering mean plasma total homocysteine [371]. In chronic schizophrenia patients, homocysteine levels declined with the addition of B vitamins, resulting in improved cognitive functions and clinical presentations [372]. Importantly, the CO concentrations required to suppress CBS became greater in the presence of SAM [344]. This study posits that vitamin B12 and folate enhance the activities of enzymes required in the remethylation of homocysteine back to methionine, producing SAM, which is the allosteric activator of CBS, while vitamin B6 enhances CBS activity itself. Both effects may result in greater CBS efficiency in vitamin B complex-treated conditions, thus providing the cells with GSH. Significantly lower levels of folate and vitamin B12 in plasma were detected in medication-naïve first-episode psychotic patients [368]. In addition, the level of vitamin B12 in the frontal cortex of patients with autism and schizophrenia was significantly decreased compared with the age-matched controls [373].

## 30. Impact of High Glucose in Neuropathologies and Co-Appearing Pathologies: Impairment of Cellular Levels of GSH

Lower G6PD activity has been reported in different settings and experimental systems, including diabetic models and diabetic patients [374,375], probably as a result of high glucose acting as an inhibitor of G6PD activity. High glucose stimulates protein kinase A (PKA), which, at least in part, causes a decrease in G6PD activity and NADPH production [376]. It has been found that it is possible to normalize redox status and the activities of redox enzymes under high glucose conditions by either overexpressing *G6PD* or inhibiting PKA.

Apparently, increased glucose acts like an inhibitor of G6PD or simply as a prohibitive factor of G6PD induction, which is required for the enhanced production of NADPH for GSH regeneration in conditions of cysteine deficiency, as in HHC (Figure 10; 10–13, 19, 20).

Hence, this study suggests that, besides having the same etiology as T2DM and some liver diseases, neuropathologies, but also T2DM and liver diseases, may be triggered by hyperglycemia, characteristics of T2DM, and some liver diseases, through a decrease in GSH regeneration. Chronic hyperglycemia caused the inhibition of G6PD activity via decreased *G6PD* expression and increased G6PD phosphorylation. Hepatic and pancreatic G6PD activities in rats, as well as the NADPH/NADP^+^ ratio and GSH level, varied inversely with high sensitivity to glucose concentrations induced by streptozotocin, and directly to the insulin levels [375]. Enhanced glycemic variability is an important predictive factor for the progression of hepatic fibrosis in NAFLD [377]. The inhibition of G6PD by high glucose resulted in decreased insulin secretion due to β-cell dysfunction in human islet culture [378]. Moreover, in animals genetically predisposed to diabetes, hyperglycemia increased rates of β-cell apoptosis [379]. Higher levels of plasma glucose were reported in many neuropathologies, as well as in drug-naïve schizophrenia patients [303]. The patients with T2DM had an increased co-appearance of dysthymia, mood disorder with psychotic symptoms, and suicidal ideation [380]. The same patients displayed an association between psychotic symptoms and higher levels of glycosylated hemoglobin, the measure of blood glucose level, as well as between dysthymia and high blood glucose. It has been shown that G6PD activity and expression, together with NADPH and GSH levels, decreased in rat kidney cortex during streptozotocin-induced diabetes [381]. On the other hand, diabetes-like features and increased aldosterone are the acquired forms of G6PD deficiency, leading to decreased NADPH and GSH levels and damage to kidney tissue and endothelial cells [382].

High glucose was found to trigger high *HMOX1* expression in pancreatic islets [383]. Increased levels of HO-1 were also found in the plasma of individuals with impaired glucose regulation, including individuals with impaired fasting glucose and impaired glucose tolerance [384]. Hyperglycemia exacerbated mitochondrial defects, i.e., decreased mitochondrial respiratory rate and caused heightened synaptic injury and cognitive dysfunction, but also resulted in a decline in hippocampal long-term potentiation reduction and spatial learning and memory in the brains of transgenic diabetic AD mice overexpressing amyloid-β [385]. Moreover, GSH may rescue VL-17A liver cells from hyperglycemia-mediated injury by reducing oxidative stress and toxicity [386].

This study posits that GSH deficit in neuropathologies may be the result of the inhibition of CBS caused by CO produced by induced HO-1 content in concert with the inhibition of G6PD caused by high glucose produced by islet and hepatic iron overload (Figure 10; 9–13, 15–19). Indeed, pathologies including porphyria, AD, PD, and HD have been associated with a deficit in GSH [387,388]. Transcripts related to an enzyme acting in GSH synthesis and metabolism were found to be increased in the prefrontal cortex of schizophrenia patients [389]. However, total GSH and the reduced GSH levels were significantly decreased in both schizophrenia and bipolar disorder patients in comparison with the healthy control subjects [390]. Postmortem studies of the brain of schizophrenia patients have shown nearly 40% depletion of GSH in the caudate nucleus [391] and reduced levels of GSH in the prefrontal cortex [392]. In addition, in drug-naïve schizophrenia patients, the levels of GSH were reduced by 52% in the prefrontal cortex and 27% in cerebrospinal fluid [393]. Moreover, in drug-free schizophrenia patients and in chronically medicated patients, the level of plasma GSH was significantly decreased and this decrease was correlated with the severity of symptoms [394].

By lowering cellular GSH, the levels of HO-1 mRNA increased approximately 11.5-fold [249]. In addition, the treatment of rats with a selective inhibitor of GSH synthesis caused an increase in HO-1 mRNA in the brain [248]. The administration of S-allylcysteine to streptozotocin-induced diabetic rats whose levels of glucose, iron, ferritin, bilirubin, and HO-1 were increased, whereas the levels of insulin, transferrin, and ALAD activity were decreased, resulted in a decrease in blood glucose, iron, ferritin, bilirubin, and HO-1 [395].

In addition, high consumption of fructose is reported to be a risk factor in the development of NAFLD [396]. Fructose upregulated hepatic gluconeogenesis by increasing corticosterone, which further activated the glucocorticoid receptor (GR) in liver, triggering GR binding to the phosphoenolpyruvate carboxykinase coding gene (*PEPCK*) [397]. PEPCK is the key regulatory enzyme in gluconeogenesis and its gene promoter has a glucocorticoid receptor-binding site. In gluconeogenesis, glucose that may act on the inhibition of G6PD is produced (Figure 10; 14, 10, 11). Interestingly, rats fed on a high-fat, high-fructose diet exhibited increased hepatic iron content [398]. A high-fat, high-fructose diet triggered reduced liver G6PD activity when compared with a low-fat, no-fructose diet [399]. Moreover, a high-fructose diet triggers ER stress and lipid accumulation. In addition, autophagy was found to alleviate hepatic ER stress in mice fed a high-fructose diet [400]. Furthermore, macroautophagy is a characteristic feature of affected neural tissues [401,402].

## 31. CYP2E1 Overproduction as a Model

In a hepatoma cell line in which *CYP2E1* was overexpressed by transfection of its expression vector, both ALAS1 and HO-1 mRNA levels were found to be markedly upregulated [403]. The treatment of cells with an inhibitor of ALAD resulted in a further increase in ALAS1 but a decrease in HO-1 mRNA levels, probably since the heme required for the induction of *HMOX-1* transcription was deficient due to the impairment of heme synthesis. Moreover, in *CYP2E1* transgenic mice, *HMOX-1* gene transcription and protein content and activity were induced while oxidative damage was enhanced [404]. Iron was cytotoxic to cells that overexpressed *CYP2E1* and not to the control cells lacking detectable CYP2E1 [405]. HepG2 cells overexpressing *CYP2E1* in the ER or mitochondria were more sensitive to GSH depletion than the control HepG2 cells without overexpressed *CYP2E1* [406]. This study argues that highly induced *CYP2E1* expression may trigger CPR saturation. Cytochrome b5 strongly stimulated CYP2E1 activity [407], implying the additional requirement for CPR reduction and, consequently, an additional requirement for GSH reducing power under the CYP2E1-overproduced condition. CYP2E1 polymorphisms were markedly associated with susceptibility to schizophrenia [408]. Indeed, in mice, ferulic acid attenuated the acetaminophen-induced increase in CYP2E1 mRNA and consequent hepatotoxicity through downregulating the increase in CYP2E1 mRNA [409]. Ferulic acid also enhanced the activities of SOD and catalase as well as the contents of GSH.

Ethanol prolonged the half-life of the protein moiety of CYP2E1 several-fold, amounting to 38 h [410,411]. Moreover, ethanol treatment was found to markedly induce HO-1 content in human hepatocytes [412]. CYP2E1 is induced by ethanol [413]. Ethanol-induced steatosis and oxidative stress has been shown to be blunted in *CYP2E1* knockout mice while it was restored in *CYP2E1* knockin mice [414]. Moreover, ethanol resulted in hepatic iron overload in human alcoholics [415]. Hepatic mitochondrial DNA was extensively damaged and degraded in ethanol-treated mice due to a decline in the mitochondrial membrane potential and opening of the mitochondrial permeability pore [413]. Recent results pointed to ethanol-induced upregulation of the miR-214 expression that is involved in the downregulation of the glutathione reductase and CPR protein levels and activities resulting in the induction of oxidative stress in human and rat liver cells, as well as in ethanol-fed Wistar rats in the liver [416]. Another study found that the depletion in cellular GSH was a key detrimental event contributing to alcoholic liver disease [413]. This study posits that deficiencies in cellular CPR and GSH contents, in concert with induced *CYP2E1* expression incurred by ethanol, result in enhanced heme release due to the disruption of holo-CYP2E1 assembly and, consequently, HO-1 induction. Induced HO-1 maintains attenuation of the heme negative feedback loop, resulting in iron overload. Increased CYP2E1 activity triggered an increase in insulin resistance, a decrease in insulin signaling in the liver, and an increase in hepatic lipid accumulation [417].

## 32. Detrimental Effect of Increased Ferritin Levels

Meta-analysis studies indicate a significant association between ferritin levels and the risk of T2DM [418,419]. In liver biopsies of AIP, the presence of numerous ferritin granules was observed [420]. In patients with NASH, that is, those histologically characterized by macrovesicular steatosis and lobular hepatitis with necrosis or ballooning degeneration and fibrosis, *HMOX1* expression was significantly increased and this increase reflected the severity of the disease [421]. In the same study, a significant correlation was observed between the increased levels of HO-1 and ferritin and between the increased levels of HO-1 and lipid peroxidation. Ferritin is elevated in AD brain tissue and cerebrospinal fluid and it predicts the conversion of mild cognitive impairment to AD [422,423]. *APOE-ε4* carriage elevates the level of cerebrospinal fluid ferritin. Interestingly, iron released by the degradation of hemin did not induce ferritin expression in cultured mouse astrocytes, whereas non-hemin free ferric iron induced ferritin expression [424]. In addition, ALA-dependent release of iron increased with an increase in human ferritin level in vitro [9].

## 33. Heme Release in Methemoglobinemia, Sickle Cell Anemia, and β-Thalassemia

In erythropoietic cells, heme is produced in order to be incorporated into hemoglobin. Two pathways of heme synthesis for the sake of the incorporation of heme b into hemoglobin and P450 are equal, with the exception of the first enzyme, which is different in these two pathways. Hence, it is of interest to explore the possibility of the same influence of released hemin from hemoglobin and P450s. Indeed, all three hemoglobinopathies—methemoglobinemia, sickle cell anemia, and β-thalassemia—are characterized by the existence of oxidized Fe^3+^ in the heme of hemoglobin; moreover, in all three disorders, heme is released, the expression of HO-1 is enhanced, and there is an excessive iron overload and mental disturbances [425,426,427,428,429,430,431,432]. Hemin (Fe^3+^ in heme) is bound less tightly by hemoglobin than heme (Fe^2+^) and it interacts more readily with acceptors such as other proteins or lipid membranes in cells [433,434,435]. Methemoglobinemia is caused by deficiency of the methemoglobin reductase enzyme, sickle cell anemia is caused by the existence of the β6 Glu-Val mutation in hemoglobin, while β-thalassemia is a result of the existence of the β26 Glu-Lys mutation in hemoglobin.

Hemoglobin (HbA) undergoes auto-oxidation (Fe^2+^ → Fe^3+^) and autoreduction (Fe^4+^ → Fe^3+^) spontaneously. Methemoglobin (ferric hemoglobin) causes an increase in HO-1 expression and elevated ferritin expression in guinea pigs, cultured endothelial cells, smooth muscle cells, and cortical glial cultures due to the release of hemin from hemoglobin species [430,434,436,437,438,439]. Methemoglobin also induced lipid peroxidation, which is, again, probably due to the activity of released hemin [430,439]. In mouse lung epithelial cells, ferric and ferryl hemoglobin induced higher expression of HO-1 than ferrous hemoglobin did, pointing to enhanced hemin release from ferric and ferryl hemoglobin species in comparison with ferrous hemoglobin species [431]. Importantly, induced HO-1 has been found in mitochondria [431,438]. Moreover, ferric and ferryl hemoglobin induce ferritin synthesis compared with ferrous hemoglobin, and they decrease the mitochondrial oxygen consumption rate [431].

A single amino acid variant of hemoglobin that is linked to severe pathology, including sickle cell Hb (HbS) (β6 Glu-Val) and β-thalassemia Hb (HbE) (β26 Glu-Lys), is amenable to higher rates of auto-oxidation of iron in the heme prosthetic unit [432,440]. Specific oxidation of βCys93 with subsequent heme loss is a marked characteristic of ferryl forms of HbS and HbE [440,441]. The β6 Glu-Val mutation in HbS leads to the accumulation of long fibers of deoxyHb molecules within red blood cells. After mixing HbO_2_ with excess H_2_O_2_, the accumulation of ferryl intermediate in HbS is almost 2.3-fold higher than that of HbA, while treatment with 5 M of excess H_2_O_2_ resulted in a two-fold higher level of Cys-93 oxidation for the HbSβ subunit compared with HbA. Treatment of ferrous forms of HbE and HbS on human pulmonary arterial endothelial cells for 24 h revealed a higher loss of endothelial monolayer integrity and lipid peroxidation [442], higher expressions of HO-1 accompanied by a rise in uncoupled mitochondrial respiration, and a higher loss of respiratory chain complex activities in isolated endothelial mitochondria and basal oxygen consumption than of HbA [432], probably all as a consequence of heme release [443,444]. Indeed, loss of membrane permeability was diminished in part by haptoglobin. The inhibition of HO-1 was found to ameliorate anemia and reduce iron overload in a β-thalassemia mouse model [445].

Hemoglobin transforms quite readily with time from its normal form to methemoglobin. It has been shown that methemoglobin forms fibers similar to ones formed with deoxyHbS in sickle cell anemia patients [446,447]. It appears that hemin in the ferric form of hemoglobin is released with time and that this hemin oxidizes cellular components, including hemoglobin molecules themselves, thus producing long filaments of deoxy-hemoglobin. In alcoholics, the levels of catalase, G6PH, and GSH were shown to be reduced while, importantly, methemoglobin as well as lipid peroxidation and protein carbonylation were increased compared with controls [448]. This may point to a similar mode of action of hemin on P450 and hemoglobin species. Moreover, in G6PD-deficient mice exposed to α-naphthol in which the GSH level was decreased, hematocrit and hemoglobin were decreased, while metHb was increased compared with the control mice [449].

## 34. Amyloid β Mimics P450 Filaments

Heme binds amyloid β (Aβ) [450]; it has been shown that increased levels of hydrogen peroxide and lipid peroxides accumulate in cells treated with Aβ and that catalase protects cells from Aβ toxicity in an Aβ concentration-dependent manner [451]. DPI inhibits Aβ toxicity in cells and inhibits hydrogen peroxide production. DPI is otherwise an inhibitor of CPR activity. Interestingly, neuroblastoma cells showed the induction of CPR upon exposure to Aβ, while tissue sections from brains of transgenic mice exhibited strong immunoreactivity for CPR surrounding Aβ [452].

The three amino acids in the N-terminal part of Aβ are decisive in activating Aβ toxicity, possibly due to heme binding [453]. It was suggested that heme binds in a 2:1 ratio in respect to Aβ and that the inherent peroxidase activity of Aβ bound with hemes is responsible for Aβ fibrillation [124]. Aβ fibrils bind free heme and AD has been suggested to represent a thrombohemorrhagic disorder [454]. It was suggested that Aβ-derived accumulation of erythrocytes in the brain tissue may lead not only to the accumulation of free heme for Aβ-heme complex formation but also to higher hydrogen peroxide concentrations due to the weaker catalase activity influenced by Aβ [455].

## 35. Ferroptosis

Ferroptosis represents a lipid peroxidation-mediated form of regulated cell death dependent of iron accumulation [456]. It occurs in diabetic osteoporosis [457], in metabolic dysfunction-associated steatotic liver disease [458], in Friedreich’s ataxia [459], and as a response to toxic effects of mycotoxins [460]. Ferroptosis also occurs in neurodegenerative diseases, particularly AD, PD, Huntignton’s disease, multiple sclerosis and ALS [461] as well as in schizophrenia [462].

It has been shown that membrane rupture and irreversible cell death induced by ferroptosis is caused by oxidation of phospholipids [463]. Polyunsaturated lipids which are constituents of biological membranes and susceptible to lipid peroxidation are essential for the ferroptosis process [464]. In the ferroptosis of polyunsaturated fatty acids (PUFAs) that are the part of phospholipids, they are stripped of their hydrogen atoms depending on iron [465] and form phospholipid radicals (PL•) which subsequently bind to oxygen molecules to form free radicals (PLOO•) [457]; hence, a reaction similar to the oxidative action of hemin on H_2_O_2_ produced by the uncoupling reaction of P450 is described in this study. It has been shown that the mitochondrial membrane or the mitochondrial and ER membranes are linked with ferroptosis [466,467].

Ferroptosis is catalyzed by CPR [468] and CYB5R1 enzymes where electron transfer activity of CPR is required for ferroptosis induction [465]. In the same study it was shown that CPR and CYB5R1 produce H_2_O_2_ and that the same two proteins induce lipid peroxidation and membrane rupture. The dependance on CPR and the production of H_2_O_2_ is in the origin of P450 oligomer production described in this study. Furthermore, it has been shown that mitochondria-depleted cells were less prone to ferroptosis [469,470]. In *FDXR*, partially deficient cells cited in this study from humans with mitochondriopathy among which is optic atrophy, neurological dysfunction, movement disorder, hearing loss, development regression, and global development delays [264,471], mitochondrial iron accumulation is associated with increased susceptibility to ferroptosis [472].

Furthermore, excessive or prolonged HO-1 activation causes iron accumulation, driving ferroptosis in atherosclerosis [473] and vascular dementia [474]. Upregulation of HO-1 in protein level in response to hemin promoted ferroptosis and caused elevation in iron level while excessive labile iron levels could in turn dramatically enhance HO-1 protein expression in ferroptotic cells in retinal pigment epithelium degeneration [475]. Treatment of cancer cells with drugs also induced HO-1-dependent ferroptosis in relation to iron accumulation [476,477,478], implicating P450 in detoxification. The difference in the effects of low to moderate and strong HO-1 expression levels on iron accumulation are responsible for cytoprotective effects and augmentation of oxidative damage due to iron accumulation, respectively [479]. In addition, a drop in GSH may trigger ferroptosis. It has been shown that GSH depletion promotes ferroptosis in cancer cells [480].

It has been shown that the Aβ oligomers accumulation has an impact on the extent of lipid peroxidation within the brain [481,482]. Inhibition of ferroptosis can result in the prevention of Aβ-like aggregates and in the attenuation of Aβ-related lipid peroxidation [483]. Furthermore, abnormal phosphorylation of Tau protein is linked with ferroptosis and lipid peroxidation in a vicious circle [484].

In mice, amyloidogenic APP processing and Aβ aggregation can be inhibited by deferoxamine (DFO), an iron chelator [485,486]. Ferroptosis can be prevented by DFO [466].

It has been shown that CYP2E1 and P4501A cytochromes trigger lipid peroxidation [487,488,489]. Hence, it could be concluded that P420 oligomerization and the production of hydroxyl radicals by P450 together with the released hemin from P420 is the triggering event of Aβ oligomers and phosphorylated Tau protein tangle production and their binding of hemin and further CPR-dependent uncoupling production of H_2_O_2_ by released hemin which results with the production of HOO^•^ in a vicious circle. Apparently, lousy binding of unreduced hemin by Aβ oligomers and Tau proteins further induces HO-1 degradation of heme inducing ALAS1 production of δ-ALA that acts on ferritin and in that way ferritin releases Fe3^+^-saturating CPR and/or adrenodoxin reductase, further enhancing the production of Aβ aggregates and neurofibriallary tangles in a vicious circle.

## 36. Conclusions

The findings of this study suggest that partial deficiency in, or saturation of, CPR, adrenodoxin, or adrenodoxin reductase activity triggers hemin release. Free hemin in concert with hydrogen peroxide produced in-cell results with the oxidation of P450 enzymes and their oligomerization, resulting in pronounced hemin release and the enhanced oxidation of other cellular components, which are responsible for neuropathologies. This study proposes that partial deficiency in, or saturation of, CPR, adrenodoxin, or adrenodoxin reductase activity may be provoked by highly increased apo-P450 content, or by some inhibitors of the P450 active site involved in their binding and activity (drugs, toxic compounds, and medications), or by some mutations in the P450 active site involved in CPR binding and activity, or by CPR, adrenodoxin, or adrenodoxin reductase mutation itself. Indeed, during treatment of CYP3A4 with podophyllotoxin and CYP2D6 with 3,4-methylenedioxymethamphetamine, both mechanism-based inhibitors of cytochromes, P420 species appear with time which, this study suggests, may be responsible for acute liver damage provoked by these two toxins [490,491]. Enhanced hemin release due to P420 oligomer creation provokes HO-1 induction. Long-term HO-1 induction may maintain the attenuation of the heme negative feedback loop over ALAS1. Moreover, when in concert with the induction of *ALAS1* expression, it may result in iron overload, mitochondrial dysfunction, and ER stress due to oxidative damage provoked by the released hemin, thus resulting in the development of neuropathologies. This study posits that, after reaching a specific level, iron overload may trigger neuropathologies having a CPR-, adrenodoxin-, or adrenodoxin reductase-saturating role. Hemin acts on H_2_O_2_ produced in the uncoupling reaction during the reduction in hemin bound in P450 resulting in the production of hydroxyl radical that causes oligomerization of P420 as well as lipid peroxidation and may damage other cellular components.

**Figure 11:**
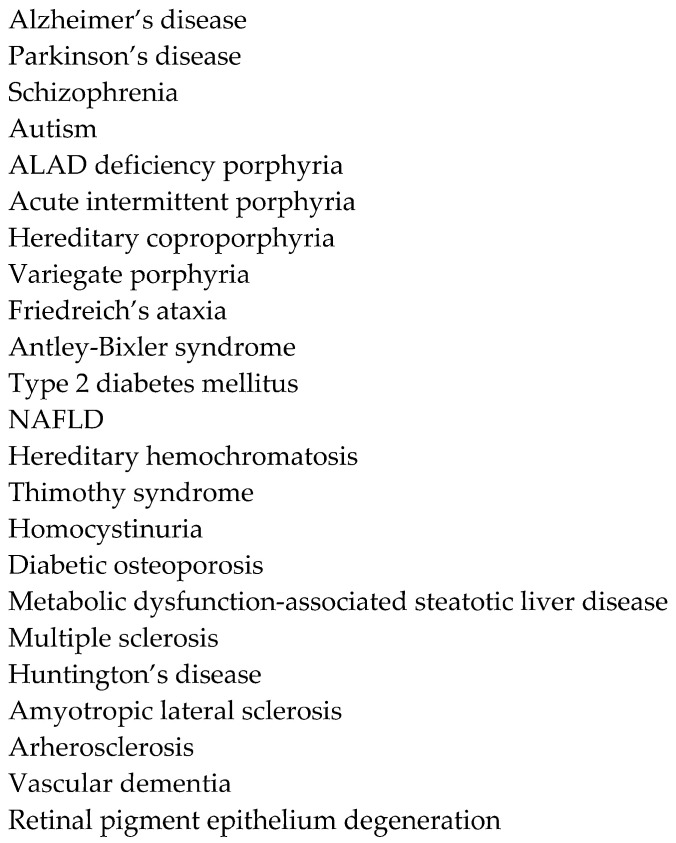
**Neuropathologies and neuropathology-linked diseases caused by P450 enzymes and cited in this article.**

Oligomers are monomerized by the action of GSH, which breaks disulfide bonds in cysteines, thus becoming susceptible to CPR action. In order to keep cytochrome heme iron in a reduced state in microsomes, one should assume the continuous requirement for the presence of GSH and CPR. The released CO produced by HO-1 may inhibit CBS activity, resulting in HHC and, in the case of increased glucose and, consequently, decreased NADPH levels, may result in reduced GSH production, and, hence, in the accumulation of unreduced cytochromes due to the appearance of oligomers that are less susceptible to CPR activity than monomers, but are susceptible to hemin release and consequent HO-1 induction. An increase in glucose triggers the inhibition of G6PD, which is the main producer of NADPH, and prevents GSH regeneration under HO-1-induced conditions. Furthermore, an increase in glucose may also be triggered by an increase in glucocorticoids. Therefore, the results of this study posit that partial CPR, adrenodoxin, or adrenodoxin reductase deficiency or saturation, as well as the faulty repair of oxidative damage produced by free hemin, along with faulty autophagy, are underlying causes of several neuropathologies and neuropathology-linked diseases (Figure 11).

Hence, P450s are involved in xenobiotic metabolism (drugs, medications, toxins, opioids, steroids) and steroid production among which are glucocorticoids, all the predisposing conditions for some neuropathologies. Although P450s undergo inactivation into P420s even without a substrate present, they require induced P450 enzymes in the cell which are induced by above-mentioned conditions. The induction of P450s is also required for the production of peroxide in the process of uncoupling which is a prerequisite for the development of neuropathologies that are induced with P450 holoenzyme metabolism.

## Data Availability

The original contributions presented in this study are included in the article. Further inquiries can be directed to the corresponding author.
